# Amyloid-β accumulation in human astrocytes induces mitochondrial disruption and changed energy metabolism

**DOI:** 10.1186/s12974-023-02722-z

**Published:** 2023-02-20

**Authors:** Marlena Zyśk, Chiara Beretta, Luana Naia, Abdulkhalek Dakhel, Linnea Påvénius, Hjalmar Brismar, Maria Lindskog, Maria Ankarcrona, Anna Erlandsson

**Affiliations:** 1 Department of Public Health and Caring Sciences, Molecular Geriatrics, Rudbeck Laboratory, Uppsala University, 752 37 Uppsala, Sweden; 2 Department of Neurobiology, Care Sciences and Society, Division of Neurogeriatrics, BioClinicum, Karolinska Institutet, 171 64 Stockholm, Sweden; 3 Science for Life Laboratory, Department of Women’s and Children’s Health, Karolinska Institutet, 171 65 Stockholm, Sweden; 4 Science for Life Laboratory, Department of Applied Physics, Royal Institute of Technology, Solna, 171 65 Stockholm, Sweden; 5 Department of Medical Cell Biology, BMC, Uppsala University, 751 23 Uppsala, Sweden

**Keywords:** Alzheimer’s disease, Glia, Lipid droplets, Mitochondria dynamics, DRP-1

## Abstract

**Background:**

Astrocytes play a central role in maintaining brain energy metabolism, but are also tightly connected to the pathogenesis of Alzheimer’s disease (AD). Our previous studies demonstrate that inflammatory astrocytes accumulate large amounts of aggregated amyloid-beta (Aβ). However, in which way these Aβ deposits influence their energy production remain unclear.

**Methods:**

The aim of the present study was to investigate how Aβ pathology in astrocytes affects their mitochondria functionality and overall energy metabolism. For this purpose, human induced pluripotent cell (hiPSC)-derived astrocytes were exposed to sonicated Aβ_42_ fibrils for 7 days and analyzed over time using different experimental approaches.

**Results:**

Our results show that to maintain stable energy production, the astrocytes initially increased their mitochondrial fusion, but eventually the Aβ-mediated stress led to abnormal mitochondrial swelling and excessive fission. Moreover, we detected increased levels of phosphorylated DRP-1 in the Aβ-exposed astrocytes, which co-localized with lipid droplets. Analysis of ATP levels, when blocking certain stages of the energy pathways, indicated a metabolic shift to peroxisomal-based fatty acid β-oxidation and glycolysis.

**Conclusions:**

Taken together, our data conclude that Aβ pathology profoundly affects human astrocytes and changes their entire energy metabolism, which could result in disturbed brain homeostasis and aggravated disease progression.

**Supplementary Information:**

The online version contains supplementary material available at 10.1186/s12974-023-02722-z.

## Introduction

Alzheimer’s disease (AD) is a progressive neurodegenerative disorder with complex pathophysiology that develops over decades. The main characteristics of AD are extracellular plaques, consisting of aggregated amyloid-beta (Aβ), intracellular neurofibrillary tangles, composed of hyperphosphorylated tau and chronic neuroinflammation [1]. The impact of glial cells in AD pathology has recently received much attention, but many questions remain regarding their involvement in disease progression. Being the most abundant glial cell type, astrocytes play an important role in maintaining brain homeostasis. Their responsibilities include recycling of neurotransmitters, modification of synapse signaling, blood–brain barrier regulation and glymphatic clearance [2–6]. In addition, astrocytes play a fundamental role in energy metabolism, and neurons, which have a very fast energy turnover, totally rely on astrocytic metabolites for their vitality.

The major cellular energy pathways include glycolysis, citric acid cycle followed by oxidative phosphorylation (OXPHOS) and fatty acid β oxidation [7–9]. Because of their anatomical position, between capillaries and neurons, astrocytes constitute an interface for effective glucose uptake from the blood. The glucose is then stored as glycogen or metabolized to pyruvate in the glycolysis. Astrocytes are highly glycolytic cells and produce lactate that is utilized by the neurons in their energy production. Moreover, astrocytes can store fatty acids that may be used as an energy source via fatty acid β oxidation [10].

Under healthy brain conditions, the metabolism of glucose and lipids is precisely regulated. Disturbed energy supply, lipid droplet (LD) formation and mitochondrial dysfunction have been linked to brain aging and neurodegenerative disorders, including AD [11, 12]. However, whether the alterations in energy supply constitute a cause or a consequence of the pathology remains unclear, as well as the underlying cellular mechanisms. Due to their significant role in energy metabolism, astrocytes are particularly interesting in this context.

Astrocytes with high Aβ load are frequently found in the human AD brain [13–17]. We have previously reported that cultured astrocytes engulf large amounts of aggregated Aβ, but then store, rather than degrade the ingested material [18–20]. The astrocytic accumulation of Aβ results in severe cellular stress and the release of extracellular vesicles (EVs) containing neurotoxic content, which could be of relevance for AD progression [18, 20]. Moreover, we have shown that astrocytes transfer protein aggregates to neighboring cells via thin protrusions called tunneling nanotubes (TNTs) [21]. Interestingly, astrocytes also forward major histocompatibility complex class II (MHCII) molecules using the same mechanism, indicating spreading of inflammation in addition to toxic protein aggregates [22]. However, in which way Aβ storage affects the astrocytes energy production remains unclear. The aim of the present study was to investigate the impact of Aβ pathology on mitochondria functionality and energy metabolism in human induced pluripotent cell (hiPSC)-derived astrocytes using a battery of different methods, with the intention to fill this knowledge gap.

## Methods

### Production of amyloid-beta fibrils

Amyloid-beta preformed fibrils were generated using human Aβ_42_ monomers (Innovagen, SP-BA42-1) and HiLyte™ Fluor 555-labeled Aβ_42_ monomers (AnaSpec, 60480–01). The Aβ monomers were dissolved in a 10 mM NaOH/PBS solution to a concentration of 2 mg/ml. The Aβ samples were left to aggregate on a shaker at 1500 rpm, 37 °C for 4d. Then, the Aβ_42_ fibrils were diluted using peptide PBS to the final concentration of 0.5 mg/ml and sonicated in 20% amplitude, 1 s off and 1 s on, for 60 s using a Sonics Vibra Cell sonicator prior to experiment. For transmission electron microscopy (TEM), Aβ_42_ fibrils or sonicated fibrils diluted 1:10 in Milli-Q water were dropped onto carbon-coated 200-mesh copper grids, negatively stained with 2% uranyl acetate for 5 min and air dried. The samples were analyzed using a Tecnai G2 transmission electron microscope (FEI Company).

### Culture of human iPSC-derived astrocytes

Human astrocytes were generated from neuroepithelial-like stem (NES) cells, produced from human induced pluripotent stem cells (iPSCs, Cntrl9 cell line) [23, 24]. The cells were cultured in Advanced DMEM/F12 (ThermoFisher, 12634–010) supplemented with 1% penicillin streptomycin (ThermoFisher, 15140–122), 1% B27 supplement (ThermoFisher, 17504–044), 1% non-essential amino acids (Merc Millipore) and 1% L-glutamine (ThermoFisher, 25030–024). The following factors were added to the medium just before use: basic fibroblast growth factor (bFGF) 10 ng/ml (ThermoFisher, 13256029), heregulin β-1 10 ng/ml (Sigma-Aldrich, SRP3055), activin A 10 ng/ml (Peprotech, 120-14E) and insulin-like growth factor 1 (IGF-1) 200 ng/ml (Sigma-Aldrich, SRP3069). Additionally, 20 ng/ml ciliary neurotrophic factor (CNTF; ThermoFisher, PHC7015) was added to the medium for the last 2 weeks of culture. Cells were passaged using trypsin 0.05% EDTA 0.2 g/l (Life Technologies) and were used for experiments directly after differentiation.

### *Aβ*_*42*_* fibril exposure*

Astrocytes were exposed to 0.2 µM sonicated Aβ_42_ fibrils in astrocyte complete medium for 7 days. Control cultures received medium without Aβ_42_ fibrils. After 3 days of Aβ_42_ fibril exposure, half the volume of fresh medium was added, without removing any conditioned medium. At day 7 the cells were washed 3 times with medium and then cultured in Aβ_42_ -free medium for 0, 6 or 12 days, with addition of half volume of fresh medium every third day. The cells were fixed or lysed at 7d, 7d + 6d, and 7d + 12d after Aβ_42_ fibril addition. All medium was collected from the cultures at the different end points and stored at – 80 °C.

### Inhibitor treatment

The cultures were exposed to triacsin C, or etomoxir. Details about exposure time and concentration are found in Table 1.

**Table 1:** Inhibitor treatments

Drug	Target	Treatment time	Concentration	Company	Cat #
Triacsin C	β Oxidation	2 h	15 µM	Sigma Aldrich	T4540-1MG
Etomoxir	β Oxidation	2 h	30 µM	Sigma Aldrich	236020-5MG

### Isolation of extracellular vesicles

The conditioned culture medium from day 7d, 7d + 6d and 7d + 12d was pooled prior to ultracentrifugation. The pooled medium samples for each treatment and cell culture batch were centrifuged at 300×*g* for 5 min to remove any free-floating cells, followed by another centrifugation at 2000×*g* for 10 min to remove any remaining cell debris. The supernatants were collected and transferred to ultracentrifuge tubes and centrifuged at 135,000×*g* at 4 °C for 1.5 h to isolate EVs, including larger microvesicles and exosomes. The vesicle pellets were resuspended in 1× PBS for TEM or in ice-cold lysis buffer (20 mM Tris pH 7.5, 0.5% Triton X-100, 0.5% deoxycholic acid, 150 mM NaCl, 10 mM EDTA, 30 mM NaPyro), supplemented with a protease inhibitor cocktail (ThermoScientific, 78430) for western blot analysis.

### Immunocytochemistry

Cells were fixed with 4% paraformaldehyde (PFA) (Sigma-Aldrich, P6148) in 1× PBS for 15 min at RT and washed 3 times with 1× PBS. Prior to antibody incubation, the cells were permeabilized and blocked with 0.1% Triton X-100 in PBS with 5% normal goat serum (NGS) (Bionordika, S-1000) for 30 min at RT. Primary antibodies were diluted in 0.1% Triton X-100 in 1× PBS with 0.5% NGS and the coverslips were incubated with the antibody solution for 1 to 4 h at RT. For primary antibodies and dilutions, see Table 2. The coverslips were washed 3 times with 1× PBS and then incubated with secondary antibodies, also diluted in 0.1% Triton X-100 in PBS with 0.5% NGS for 45 min at 37 °C. The secondary antibodies used were AlexaFluor 488, 555 or 647 against mouse, rabbit or chicken (1:200, Molecular Probes). The coverslips were washed 3 times with 1 × PBS and mounted on microscope slides using EverBrite hard-set medium with DAPI (230032, Biotium). Images were captured using a fluorescence microscope Observer and Z1 Zeiss.

**Table 2:** Overview of primary antibodies

Antibody	Host	Technique	Target	Company	Cat #
Vimentin	Chicken	Immunocytochemistry (1:500)	Astrocytes	Sigma Aldrich	AB5733
GLAST-1	Rabbit	Immunocytochemistry (1:400)	Astrocytes	Novus Biologicals	NB100-1869
AQP4	Rabbit	Immunocytochemistry (1:200)	Astrocytes	Novus Biologicals	NBP1-87679
GFAP	Chicken	Immunocytochemistry (1:400)	Astrocytes	Abcam	ab4674
Nestin	Rabbit	Immunocytochemistry (1:200)	Astrocytes	Millipore	ABD69
COX IV	Rabbit	Immunocytochemistry (1:200), WB (1:500)	Mitochondria	Abcam	ab14744
VDAC-1	Mouse	Immunocytochemistry (1:200), WB (1:500)	Mitochondria	Abcam	ab14734
pDRP-1 (S616)	Rabbit	Immunocytochemistry (1:200), WB (1:500)	Mitochondrial dynamic	Cell signaling	4494S
pDPR-1 (S637)	Rabbit	Immunocytochemistry (1:200), WB (1:500)	Mitochondrial dynamic	Abcam	ab193216
DRP-1	Mouse	Immunocytochemistry (1:200), WB (1:500)	Mitochondrial dynamic	Abcam	ab56788
Catalase	Rabbit	Immunocytochemistry (1:200), WB (1:500)	Peroxisomes	Abcam	ab16731
PMP70	Mouse	Immunocytochemistry (1:200), WB (1:500)	Peroxisomes	Abcam	ab211533
Flotillin1	Rabbit	Immunocytochemistry (1:500), WB (1:500)	Extracellular vesicles	Abcam	ab41927
S100β	Mouse	Immunocytochemistry (1:1000)	Astrocytes	Sigma Aldrich	S2532
OPA1	Mouse	WB (1:200)	Mitochondrial dynamic	Beckman Dickinson	612606
BAX	Rabbit	WB (1:200)	Apoptosis	Abcam	ab199677
LDH	Rabbit	Immunocytochemistry (1:200), WB (1:500)	Glycolysis	Abcam	ab75167
Caspase-3	Rabbit	WB (1:500)	Apoptosis	Abcam	ab4051
GAPDH	Mouse	WB (1:5000)	Loading control	Abcam	ab8245

#### Dyes

To visualize actin, cover slips were incubated 30 min in phalloidin-fluorescein dye (2 µM, Sigma Aldrich, P5282).

To visualize cellular membranes, cover slips were incubated with Wheat Germ Agglutinin (WGA), Alexa Fluor™ 350 Conjugate (10 µg/ml, ThermoFisher, W11263) for 2 h at 37 °C.

To visualize neutral lipids, cover slips were incubated with BODIPY 493/503 (1:100, ThermoFisher, D3922) for 30 min at 37 °C.

### Labeling mitochondria for time-lapse imaging

For the motility analysis, cells were transfected with either CellLight Mitochondria-GFP or Mitochondria-RFP Bac 2.0 constructs at the concentration of 20 particles per cell during the last 2 days of the experiment. For the transfer analysis, cells were labeled using MitoTracker™ Green FM according to the manufacturer’s instructions.

### Time-lapse microscopy

Time-lapse experiments were performed at 37 °C in humidified 5% CO_2_ in air, using a Nikon Biostation IM Live Cell Recorder (Nikon). The cells were cultured at a concentration of 5000 cells/cm^2^ in time-lapse culture dishes (VWR) and images were captured every 12 s for 20 min for the motility analysis and every 2 min for 24 h for the transfer analysis. Movies of the time points of interest were exported at a speed of 10 frames/second.

### Transmission electron microscopy

#### EVs

EV samples were mixed with an equal volume of 4% paraformaldehyde, added onto a formvar and carbon-coated 200-mesh grid (Oxford 11 Instruments) and incubated for 20 min. After incubation, the grid was dried and washed first 3 times with PBS, followed by 8 washes in MilliQ water. The samples were stained in a drop of uranyl oxalate, pH = 7, for 5 min, after which they were stained with a drop uranyl acetate 4% pH = 4 with 2% methylcellulose on ice for 10 min. The dried grids were imaged in a Tecnai G2 transmission electron microscope (TEM, FEI company) with an ORIUS SC200 CCD camera and Gatan Digital Micrograph software (both from Gatan Inc.).

#### Cells

The cultures were fixed in 2.5% glutaraldehyde and 1% paraformaldehyde. The cells were then rinsed with 0.15 M sodium cacodylate (pH 7.2–7.4) for 10 min and incubated in fresh 1% osmium tetraoxide in 0.1 M sodium cacodylate for 1 h at RT. After incubation, the sodium cacodylate was rinsed away to dehydrate the dishes with 70% ethanol for 30 min, 95% ethanol for 30 min and > 99% ethanol for 1 h. A thin plastic layer (Agar 100 resin kit, Agar Scientific Ltd) was added to the dishes and incubated for 1 h. The plastic was poured off and a new plastic layer was added onto the dishes for incubation overnight in a desiccator. Next, the plastic was heated to enable its removal after which a new thicker plastic layer was added before another incubation for 1 h in a desiccator. Cells were covered with 3-mm plastic and polymerized in the oven at 60 °C for 48 h. Embedded cells were sectioned by using a Leica ultracut UTC ultramicrotome (Rowaco AB) and visualized with a Tecnai G2 transmission electron microscope (FEI company) with an ORIUS SC200 CCD camera and Gatan Digital Micrograph software (both from Gatan Inc.).

### Cell lysis

The cell culture medium was removed and the cells were lysed in ice-cold lysis buffer (20 mM Tris pH 7.5, 0.5% Triton X-100, 0.5% deoxycholic acid, 150 mM NaCl, 10 mM EDTA, 30 mM NaPyro), supplemented with a protease inhibitor cocktail (ThermoScientific). The lysates were transferred to protein LoBind tubes (Eppendorf) and incubated for 30 min on ice prior to centrifugation at 10,000×*g* for 10 min at 4 °C. The supernatants were transferred to new tubes and stored at -70 °C until analysis. To measure the protein concentration, the supernatants were analyzed with Pierce BCA Protein Assay kit (23225, ThermoFisher).

### Immunoprecipitation

Immunoprecipitation was performed using Dynabeads Protein G Immunoprecipitation kit (10007D, ThermoFisher). Each 1.5 mg Dynabeads protein G (ThermoFisher Scientific) was bound with 5 µg of antibody (10 min, RT, gentle shaking) and incubated with 200 µl of EVs lysates (2 h at RT, gentle shaking). Protein elution was conducted with 20 µl elution buffer and 10 µl Western Blot loading buffer. After each step, beads were concentrated on magnetic particle concentrator and carefully washed with recommended washing buffer.

### Western blot

Protein concentrations of the total cell lysates were measured with Pierce BCA protein kit (ThermoFisher Scientific), according to the manufacturer’s instructions. A total of 18 μg protein was mixed with Bolt LDS Sample buffer and Sample Reducing agent (both from ThermoFisher Scientific) and incubated for 10 min at 80 °C to denature the proteins. Samples were loaded on a Bolt 4–12% Bis–Tris plus gel and run in Bolt MES sodium dodecyl sulfate (SDS) running buffer (both from ThermoFisher Scientific) for 22 min at 200 V. Chameleon duo pre-stained protein ladder (Li-Cor) was used for visualization of gel migration, protein size and orientation. Transfer to a PVDF membrane was performed for 1 h at 20 V in Bolt transfer buffer containing 10% methanol, 0.1% Bolt antioxidant (ThermoFisher Scientific) and 0.01% SDS. Blocking of the membrane was performed in 5% bovine serum albumin (BSA, Sigma-Aldrich) in 0.1% Tris-buffered saline-Tween (TBS-T) for 1 h on shake at RT, prior to incubation with primary antibody in 0.5% BSA in 0.1% TBS-T O/N at 4 °C. For specific antibodies and dilutions, see Table 2. Following extensive washes in TBS-T, the membrane was incubated with horseradish peroxidase-conjugated (HRP) secondary goat anti-rabbit (1:20,000, Sigma Aldrich) or goat anti-mouse antibody (1:20,000, Pierce) in 0.5% BSA in 0.1% TBS-T for 1 h on shake at RT. Development of membrane was performed with enhanced chemiluminescence (ECL, GE Healthcare) by using a ChemiDoc XRS with Image Lab Software to visualize and measure the intensity of the immunoreactive bands (Bio-Rad Laboratories).

### ATP and lactate levels

The cell culture medium was removed and the cells were lysed for 30 min in ice-cold 4% perchloric acid. Next, lysates were centrifuged at 10,000×*g* for 10 min at 4 °C. Immediately after centrifugation, the supernatants were used to assay ATP and lactate levels. Biochemical assays were conducted according to the manufacturer’s instruction (ATP assay kit, ab83355, Abcam; Lactate assay kit, ab65330, Abcam). To measure the protein concentration, the pellet was dissolved in 1 NaOH and analyzed with Pierce BCA Protein Assay kit (23225, ThermoFisher).

### Oxygen consumption rate (OCR) and extracellular acidification rate (ECAR) measurements

Astrocytes were plated in poly-D-coated Seahorse XF96 microplates (Agilent). The day before the experiment, the sensor cartridge (Seahorse Bioscience) was hydrated with the Seahorse XF Calibrant in a humified CO2-free incubator at 37 °C, overnight.

For OCR measurements, cell media was replaced by unbuffered DMEM supplemented with 17.5 mM glucose, 1 mM pyruvate and 2 mM L-glutamine, pH = 7.2–7.4. OCR was measured using the Seahorse® XF96 analyzer (Agilent) at baseline, and after sequential stimulation with 1 µM oligomycin A (75351, Sigma Aldrich), 1 µM carbonyl cyanide-4-(trifluoromethoxy)phenylhydrazone (FCCP) (C2920, Sigma Aldrich), and 0.5 µM antimycin A (Ant A) (A8674, Sigma Aldrich) plus rotenone 0.5 µM (Rot) (R8875, Sigma Aldrich). Accordingly, mitochondrial basal respiration, maximal respiration, ATP production and spare respiratory capacity (SRC) were automatically calculated using the Seahorse XF Cell Mito Stress test report generator from Wave 2.6.1 software (Agilent). Data were given in pmol O2/min and normalized for protein content.

For ECAR measurements, cell media was replaced by unbuffered DMEM medium supplemented with 2 mM l-glutamine. ECAR values were sequentially obtained at baseline and following sequential stimulation with 15 mM glucose, 1 µM oligomycin A, and 50 mM 2-deoxy-d-glucose (D9761, Sigma Aldrich). Glycolysis, glycolytic capacity, glycolytic reserve values were obtained using the Seahorse XF Glycolytic Stress Test Summary Report from Wave 2.6.1 software. Data were presented in mpH/min/µg protein.

### AlamarBlue test

The viability test was conducted according to the manufacturer’s instruction. Briefly, cells seeded at 96 well plate were treated with AlamarBlue dye for 2 h in 37 ˚C (1:10, ThermoFisher, 1025). The fluorescence intensity was read with 560/590 nm (excitation/emission).

### Image analysis

#### Motility analysis of CellLight mitochondria

Analysis of mitochondria motility was performed on time-lapse recordings of astrocytes transfected with CellLight Mitochondria-RFP or CellLight Mitochondria–GFP, by segmenting and tracking individual mitochondrion using the Mitometer MATLAB algorithm [25]. Time-lapse recordings with 90–180 frames at 12 s intervals were analyzed. The motility parameters displacement, distance, speed and velocity were analyzed. Displacement is the Euclidian distance between start and end position for a mitochondrion while the distance parameter expresses the accumulated length of a mitochondrion track. Velocity is the momentaneous speed, i.e., distance between two frames while the speed parameter expresses the average velocity for a track (Additional file 2A). The mean value per mitochondria over time was used to calculate the average value per cell for distance, displacement, speed and velocity.

#### Morphology analyses of CellLight mitochondria

Morphology analyses of mitochondria in astrocytes transfected with CellLight Mitochondria-RFP or CellLight Mitochondria-GFP construct were measured with MiNa ImageJ macro and quantified using Valentine and colleagues’ protocol [26]. In total, data were analyzed from 3 independent experiments (each time point and treatment was captured with 20 images (63x) per one set of experiment). Image pre-processing assessment included: image sharpening, contrast enhancing, binary image production and image skeletonization. The MiNa image processing reports branches and network was normalized to the number of analyzed cells. MiNa ImageJ macro defines an individual mitochondrion as an object that only contains 1 or 0 branches (a rod or puncta).

#### Morphology analysis of mitochondria in TEM images

For measurements of mitochondrial dynamic, the mitochondrial length and width were measured using ImageJ software in the TEM images. Only mitochondria with clearly recognizable cristae were analyzed. To receive the mitochondrial aspect ratio, the mitochondrial length was divided by the mitochondrial width.

### Statistical analysis

The Shapiro–Wilk’s normality test excluded normal data distribution. Therefore, the data were analyzed by nonparametric Mann–Whitney test, using GraphPad Prism (version 5.03) and shown as median with interquartile ranges. The level of significance for all the graphs was: *P < 0.05, **P < 0.01, and ***P < 0.001.

### Immunocytochemistry

A minimum of 60 images per time point and treatment for each culture system were captured at 63× magnification from three-five independent experiments. The IntDen fluorescence intensity signal was measured using ImageJ software.

#### Morphology analyses of CellLight mitochondria

A minimum of 60 images per time point and treatment for each culture system were captured at 63× magnification from three independent experiments.

#### Morphology analysis of mitochondria in TEM images

At least 300 mitochondria were assessed per time point and treatment for each culture system.

### Western blot

The presented WB data were calculated from 3 or 6 independent experiments.

### ATP and lactate levels

The metabolite levels were calculated from 3 or 6 independent experiments.

### AlamarBlue test

The viability test was assayed in 3 independent experiments.

### Motility analysis

600–2500 mitochondria per cell were analyzed from 11 or 12 time-lapse recordings, for control and Aβ-exposed astrocytes, respectively. Mean mitochondria displacement, distance, speed and velocity were calculated and analyzed by Wilcoxon rank test and shown in the graphs as mean with 95% confidence interval of the values for all individual mitochondrion.

## Results

### Astrocytes ingest and accumulate sonicated Aβ_42_ fibrils

**Fig. 1:**
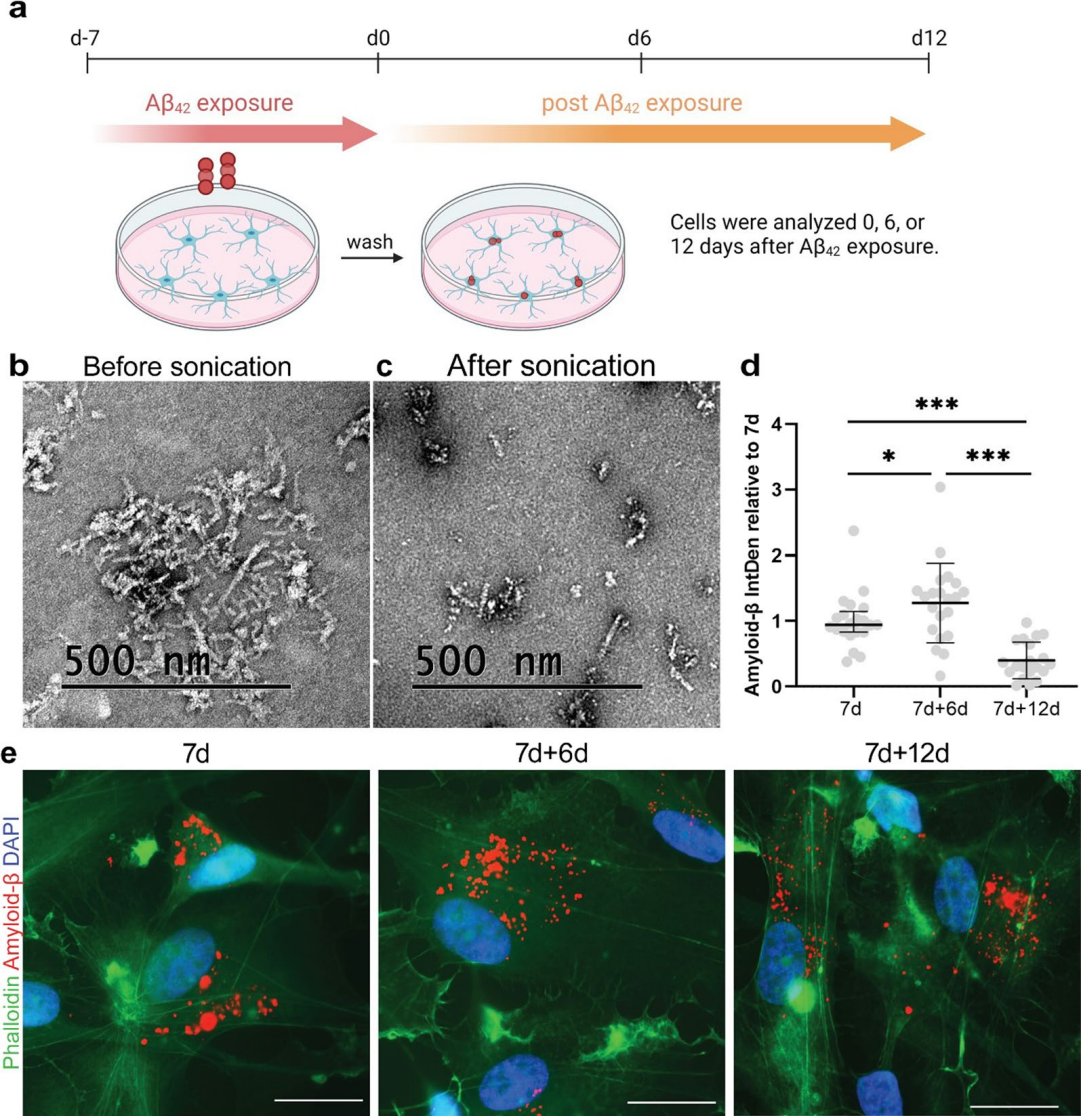
Astrocytes engulf and accumulate Aβ. Human iPSC derived astrocytes were exposed to sonicated Aβ fibrils for 7 days and the cells were analyzed 0 days (7d), 6 days (7d + 6d) or 12 days (7d + 12d) after exposure (**A**). Aβ fibrils were visualized with TEM before (**B**) and after sonication (**C**). Quantification of the HiLyteFluor555 signal, revealed that there was a slight rise in Aβ signal at 7d + 6d followed by a decrease at 7d + 12d (**D**). Aβ inclusions were found inside the astrocytes at all three time points (**E**). Scale bar: **E** = 20 µm

Human astrocytes, expressing the astrocytic markers GLAST-1, vimentin, S100β, AQP4, GFAP and nestin (Additional file 1) were exposed to sonicated HiLyte™ Fluor 555-labeled Aβ_42_ fibrils for 7 days. The astrocytes were then washed and cultured for additional 0, 6 or 12 days (referred as 7d, 7d + 6d, and 7d + 12d, respectively) in Aβ_42_ free medium (Fig. 1A). TEM analysis confirmed that the sonication of the fibrils resulted in smaller Aβ_42_ aggregates (Fig. 1B, C). In line with our previous observations that astrocytes easily degrade monomeric proteins, while accumulate larger oligomers and fibrils [18–21], we found that the astrocytes ingested the Aβ_42_ aggregates during the 7 days exposure, but did not effectively degrade the material. Hence, intracellular Aβ_42_ deposits were observed at all three time points (Fig. 1D, E). Image analysis revealed that, upon removal of extracellular sonicated Aβ_42_ fibrils, the intracellular Aβ signal initially increased (Fig. 1D). This result can be explained by the fact that the Aβ aggregates are brought closer together over the 6-day period. Upon ingestion, the aggregates are relocated inside the cell to end up in “storage dumps” around the nucleus, a phenomenon that we have previously documented in different astrocytic culture systems following exposure to aggregated Aβ or α-synuclein [20, 21, 27]. However, at day 7d + 12d the intracellular Aβ signal was reduced compared to day 7, indicating that the astrocytes were capable of degrading at least a proportion of the ingested Aβ_42_ fibrils over time (Fig. 1D).

### Accumulation of Aβ in astrocytes results in mitochondrial network alterations

**Fig. 2:**
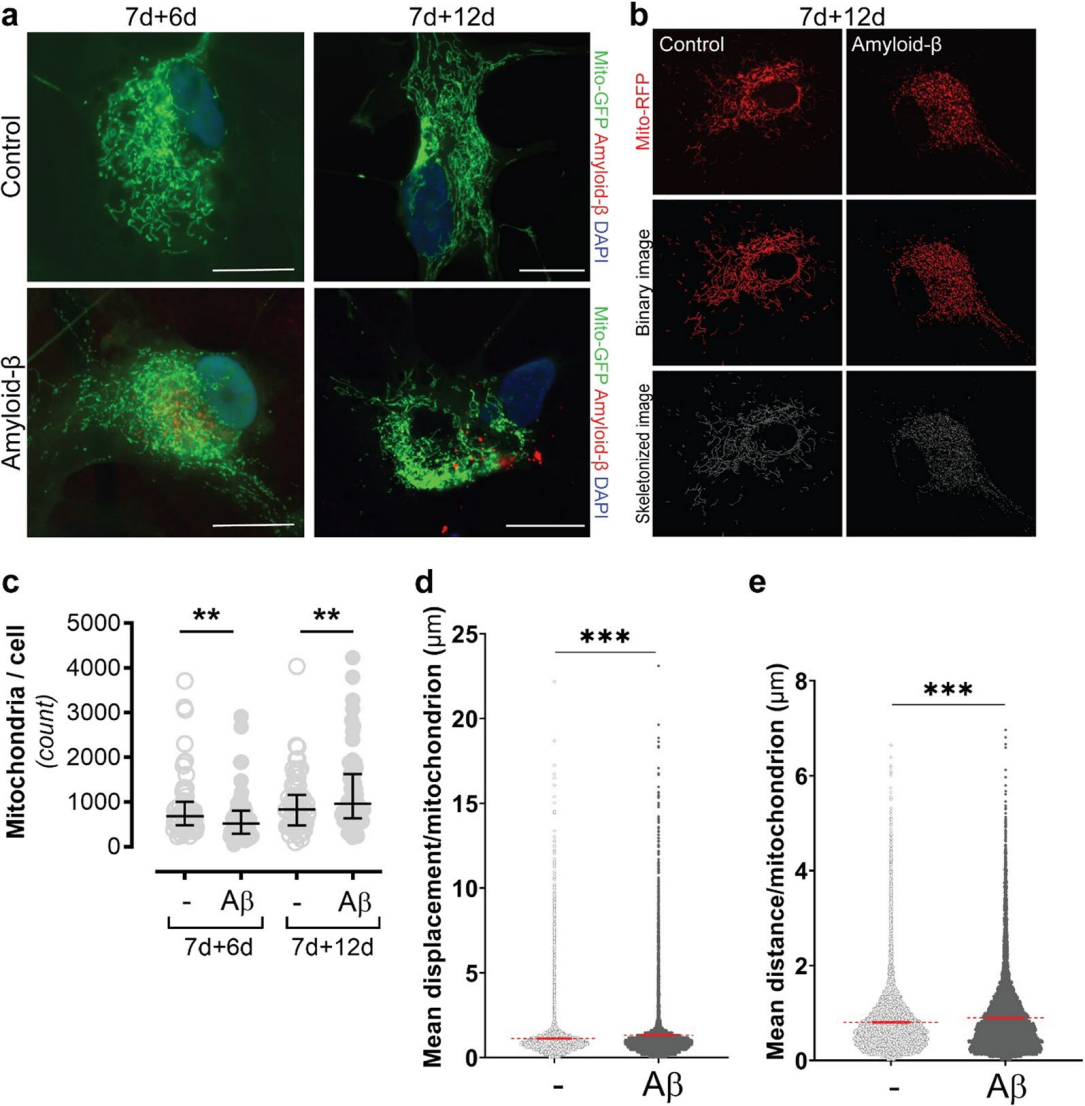
Mitochondrial networks are severely affected by Aβ accumulation. The cells were labeled with CellLight Mitochondria-GFP followed by ICC in order to visualize the mitochondrial networks (green), Aβ (red) and the cell nuclei (blue) (**A**). MiNa ImageJ macro was used to analyze the networks and the images were skeletonized for optimal analysis (**B**). The quantifications showed that there was a decrease in the number of mitochondria in Aβ-exposed astrocytes at day 7d + 6d, but an increase at 7d + 12d (**C**). Motility analysis, using Mitometer, revealed that mitochondria displacement (**D**) and distance (**E**) were increased in Aβ-exposed astrocytes. Scale bar: **A** = 20 µm

To investigate how the mitochondrial network is affected in Aβ-accumulating astrocytes, we labeled the mitochondria with CellLight Mitochondria-GFP or -RFP (Fig. 2A), and used the MiNa ImageJ macro [26] for quantification. In order to create the optimal condition for the macro, we converted the microscopy photos to binary images that were then skeletonized (Fig. 2B). Our analysis of the skeletonized images showed that the number of mitochondria per astrocyte were significantly decreased in Aβ-exposed cultures compared to control cultures at 7d + 6d (Fig. 2C). However, at the later time point, 7d + 12d, the result was the opposite, with a significant increase in the number of mitochondria per astrocyte in the Aβ-exposed cultures (Fig. 2C). Hence, the effect of Aβ-accumulation on mitochondrial number changes over time.

Next, we studied the mitochondria motility in time-lapse recordings of Aβ-exposed astrocytes and control astrocytes, focusing on the parameters; mitochondrial displacement, distance, speed and velocity [25]. Interestingly, Aβ-exposed astrocytes showed a higher mitochondrial displacement (17% increase), compared to control astrocytes (Fig. 2D). A similar pattern was found for the distance parameter (13% increase), indicating that mitochondria in Aβ accumulating astrocytes traveled longer distances, compared to mitochondria in control astrocytes (Fig. 2E). There was also a modest increase in mitochondrial speed and velocity in Aβ-exposed astrocytes (10% and 14% increase, respectively; Additional file 2B, C). Taken together, these results indicate that the change in mitochondrial number in Aβ-exposed astrocytes is entailed with an altered mitochondria motility pattern.

**Fig. 3:**
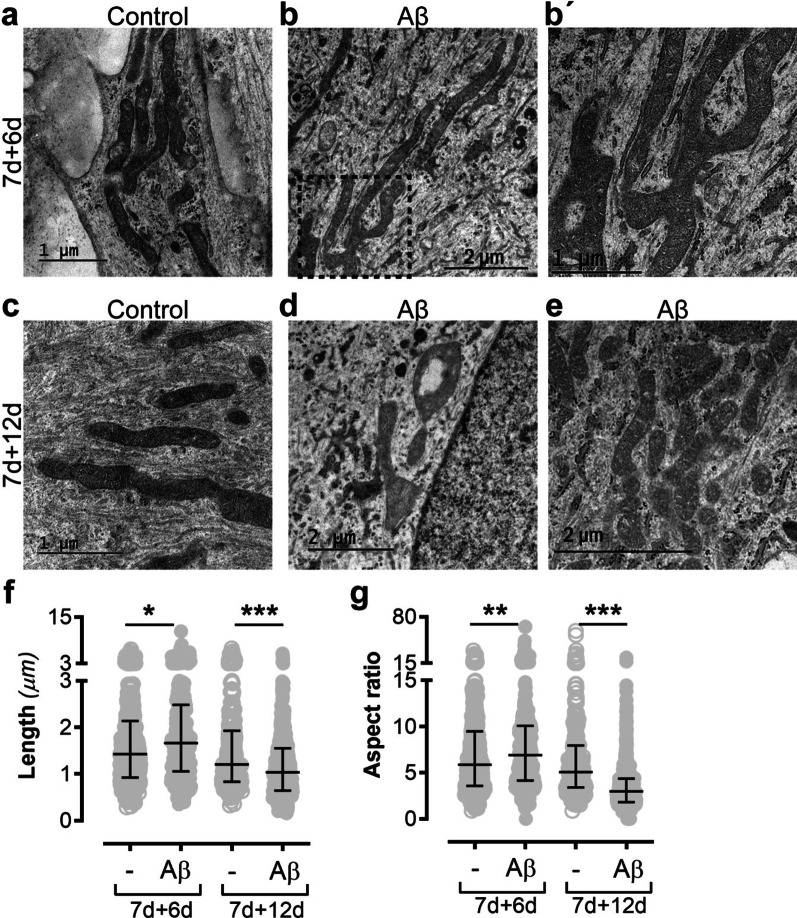
Aβ accumulation causes mitochondrial fission and fusion dysfunction. TEM analysis demonstrated that while control astrocytes had healthy mitochondria profiles at 7d + 6d (**A**), astrocytes exposed to Aβ displayed mitochondria profiles that were extremely long (**B**) and abnormally branched (**B’**). At 7d + 12, the mitochondria profiles in the control cultures still showed a healthy morphology (**C**), while in the Aβ-exposed astrocytes had swollen (**D**) and very short (**E**) mitochondria profiles. Quantifications confirmed that mitochondria profiles in Aβ-exposed astrocytes were significantly longer than in control astrocytes at 7d + 6d, but significantly shorter at 7d + 12d (**F**). The aspect ratio between mitochondria length and width showed the same pattern (**G**)

Mitochondrial fission and fusion are central processes that are important for maintaining mitochondrial health and function. In order to investigate the effect of Aβ-accumulation on fission/fusion homeostasis, we performed TEM on Aβ-exposed astrocytes and control astrocytes at two time points. At 7d + 6d, the mitochondria in control astrocytes displayed a healthy morphology, with an intact and clear cristae and no signs of swelling (Fig. 3A). In contrast, the mitochondria in Aβ-accumulating astrocytes were extremely long (Fig. 3B) and formed unusual, branched structures (Fig. 3B’), indicating exaggerated fusion or reduced fission. At 7d + 12d, the mitochondria profiles were unchanged in the controls (Fig. 3C), but in the Aβ-exposed astrocytes the mitochondria profiles were very short, severely swollen and with disrupted cristae, indicating that they had instead undergone excessive fission (Fig. 3D, E). When we quantified the length of the mitochondria profiles in the cultures, we could show that mitochondria profiles in Aβ-exposed astrocytes were significantly longer than in control astrocytes at 7d + 6d, but significantly shorter at 7d + 12d (Fig. 3F). The same pattern was observed when we quantified the aspect ratio between length and width (Fig. 3G). These results indicate that Aβ accumulation in astrocytes severely affects their mitochondria fission/fusion homeostasis.

### DRP-1 phosphorylation and expression pattern changes when the astrocytes accumulate Aβ

**Fig. 4:**
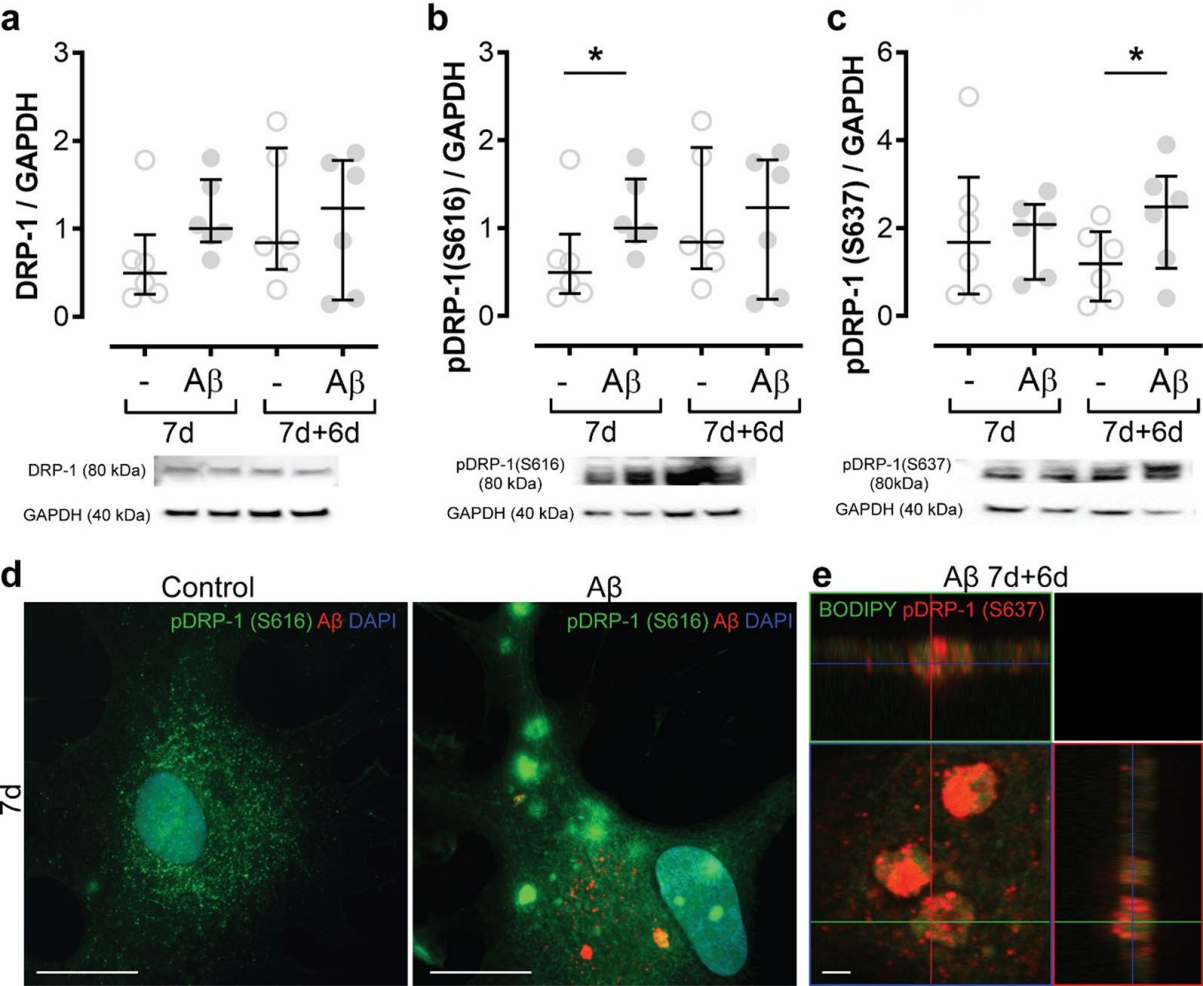
Both phosphorylation and expression pattern of DRP-1 is altered in Aβ accumulating astrocytes. To investigate fission and fusion changes further, the total DRP-1 levels were measured, but there were no changes (**A**). Depending on where DRP-1 is phosphorylated it can enhance fission (S616) or fusion/fission (S637). However, the protein may also have other functions. Levels of pDRP-1 (S616) were increased in Aβ-exposed astrocytes at day 7 (**B**), while pDRP-1 (S637) was increased at 7d + 6d after Aβ exposure (**C**). ICC revealed that the pattern of pDRP-1 (S616) changes dramatically in the presence of Aβ. Aβ-accumulating astrocytes displayed large, concentrated clumps of pDRP-1 (S616), which was not present in control astrocytes (**D**). Moreover, pDRP-1 (S637) was found to encapsulate BODIPY-positive lipid droplets (**E**). The separate channels of the merged image (**E**) are shown in Additional file 4. Scale bar: **D** = 20 µm; **E** = 2 µm

To further elucidate the changes in mitochondrial fission and fusion in Aβ-accumulating astrocytes, we next analyzed the key proteins involved in these processes. As the levels of OPA1, which is involved in mitochondrial fusion, were not affected by Aβ exposure (Additional file 3), we focused on the dynamin related protein 1 (DRP-1). Recruitment of DRP-1 to mitochondria is a central step required for mitochondrial fission, which can be triggered or blocked by phosphorylation at different sites by distinct kinases. Hence, we investigated the proportion of DRP-1 that were either phosphorylated at Ser616 (S616), which promotes mitochondrial fission, or at Ser637 (S637), which can either activate fission or induce detachment of DRP-1 from the mitochondria, depending on which kinase that is involved in its phosphorylation [28, 29]. Western blot analysis showed that the total DRP-1 levels were unchanged (Fig. 4A), while DRP-1 (S616) was increased in Aβ-exposed astrocytes at 7d (Fig. 4B) and DRP-1 (S637) was increased in Aβ-exposed cultures at 7d + 6d (Fig. 4C). Interestingly, immunocytochemistry revealed that the expression pattern of phosphorylated DRP-1 was dramatically changed in the astrocytes after Aβ exposure. In control astrocytes, DRP-1 (S616) was evenly distributed at 7d, but in Aβ-exposed astrocytes, the protein formed large clumps (Fig. 4D). At 7d + 6d the DRP-1 (S637) was particularly found around BODIPY-positive lipid droplets in Aβ-exposed astrocytes, suggesting a possible role of phosphorylated DRP-1 in lipid droplet metabolism in addition to mitochondrial fission/fusion (Fig. 4E).

### Phosphorylated DRP-1 is transferred between astrocytes via EVs and TNTs

**Fig. 5:**
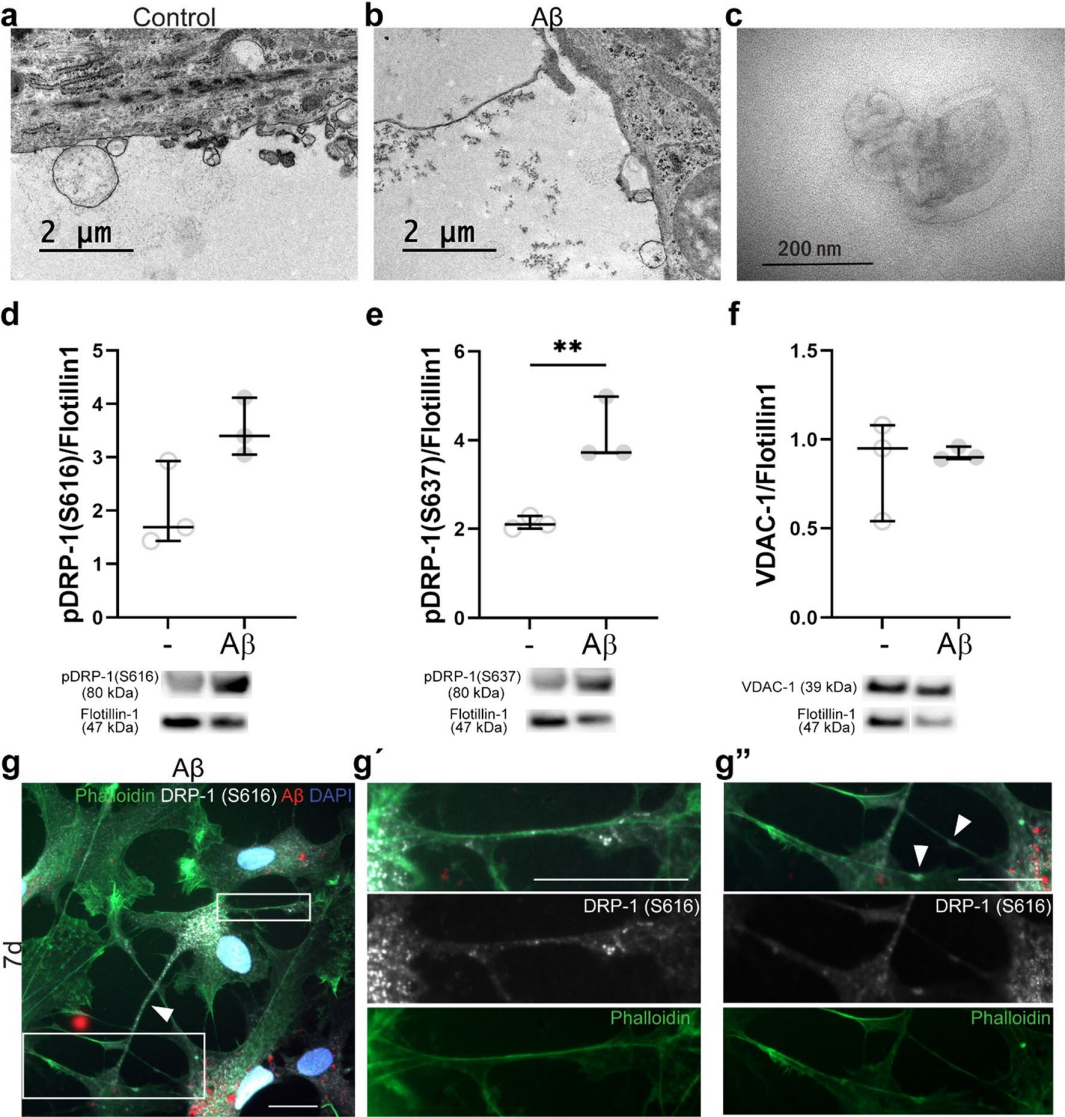
Astrocytes transfer pDRP-1 through EVs and TNT. TEM showed EVs in close proximity to the astrocytes in controls (**A**) and Aβ-exposed cultures (**B**). EVs were visualized with TEM after isolation through ultracentrifugation (**C**). WB analysis of the isolated EVs demonstrated that pDRP-1 (S616) (**D**) and pDRP-1 (S637) (**E**) are secreted from astrocytes, also showing a significant increase of pDRP-1 (S637) after Aβ exposure. However, the levels of the mitochondrial marker VDAC-1 were stable (**F**). ICC stainings of Aβ-exposed astrocytes from 7d showed the presence of pDRP-1 (S616) in both thicker protrusions (white arrowhead) (**G**) and in thin TNTs (**G’** and white arrowheads in **G”**) connecting the cells. Scale bar: 20 µm

Based on our previous data that astrocytes actively transport aggregated proteins to surrounding cells, we sought to investigate if astrocytes also secrete and/or transfer phosphorylated DRP-1 via extracellular vesicles (EVs) or tunneling nanotubes (TNTs). By TEM, we could confirm the presence of EVs around both control astrocytes and Aβ-exposed astrocytes (Fig. 5A, B), as well as in the conditioned media following ultracentrifugation (Fig. 5C). The EV fractions were analyzed with western blot analysis using antibodies towards the phosphorylated DRP-1 proteins and the mitochondrial membrane marker voltage-dependent anion-selective channel 1 (VDAC-1). The results demonstrated that both control and Aβ-exposed astrocytes secrete pDRP-1 proteins. However, there was a trend of increased pDRP-1 (S616) (Fig. 5D) and a significant twofold increase in pDRP-1 (S637) (Fig. 5E) in EVs isolated from the Aβ-exposed astrocytes, while VDAC-1 levels were not significantly changed (Fig. 5F). Hence, the increased secretion of DRP-1 proteins is not caused by the secretion of whole mitochondria. These data indicate that Aβ-exposed astrocytes are more prone to secrete EVs with pDRP-1 content than control astrocytes.

Beside EVs, astrocytes are also known to transfer organelles and aggregated proteins through TNTs. To investigate this mechanism, we performed immunocytochemistry using phalloidin to visualize filamentous actin and antibodies against pDRP-1 (S616) (Fig. 5G) as well as time-lapse microscopy of GFP-labeled mitochondria (Additional files 5 and 6). Indeed, the live imaging experiments demonstrated that mitochondria are frequently transferred from one astrocyte to another via TNTs (Additional files 5 and 6). Moreover, pDRP-1 (S616) was found in both thicker protrusions (Fig. 5G, white arrowhead) and in thinner TNTs (Fig. 5G’-G”) connecting the astrocytes, indicating that mitochondria with pDRP-1 in their outer membrane and/or deposits of phosphorylated pDRP-1 are distributed between the cells.

### Aβ accumulation in astrocytes affects OXPHOS spare respiratory capacity, despite no changes in ATP production

**Fig. 6:**
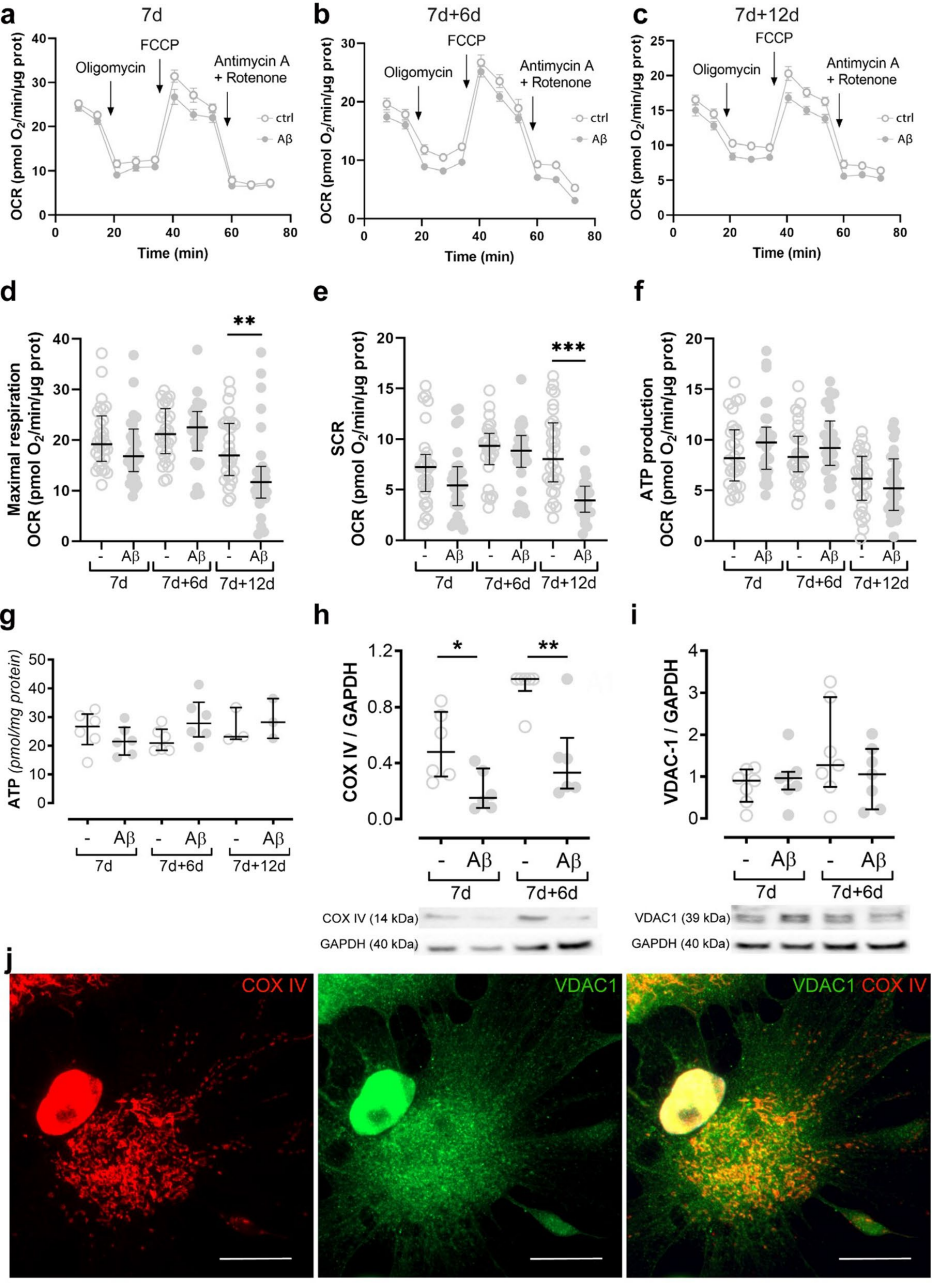
Aβ exposure affects OXPHOS in astrocytes, but the ATP levels remain unchanged. Seahorse OCR analysis was performed in astrocyte cultures exposed to Aβ and untreated controls at three different time points; 7d, 7d + 6d, and 7d + 12d (**A**–**C**). Maximal respiration and spare respiratory (SRC) capacity were significantly decreased in Aβ-exposed astrocytes compared to controls at 7d + 12d (**D**, **E**). ATP levels remained unchanged both when analyzed with Seahorse OCR (**F**) and ATP assay (**G**). WB analysis showed a clear decrease in COX IV expression in Aβ-exposed astrocytes, both at 7 and at 7 + 6 d (**H**), suggesting a reduced activity of the electron transport chain in these cultures. Since VDAC-1 levels were stable between controls and Aβ-exposed cultures (**I**), the COX IV decrease was not a result of reduced number of mitochondria. Immunostainings for COX IV (red) and VDAC-1 (green) in control astrocytes did not reveal a complete overlap of the mitochondrial markers, indicating that there is a mitochondria population in the astrocytes that is COX IV negative (**J**). Scale bar: **A** = 20 µm

To further study the impact of Aβ accumulation on mitochondrial function, we used the Seahorse flux analyzer to evaluate OCR. No changes in mitochondrial respiration were observed in 7d or 7d + 6d in Aβ-exposed astrocytes (Fig. 6A, B, D–F, Additional file 7A). However, at 7d + 12d the astrocytes showed significant deficits in maximal mitochondrial respiration and spare respiratory capacity (SRC) (Fig. 6C–E), despite no alterations on basal respiration or ATP production (Fig. 6F, Additional file 7A). Total ATP levels were also unchanged (Fig. 6G), confirming the results from the Seahorse analysis. Taken together, these data point to an impaired ability of mitochondria in Aβ-exposed astrocytes to increase OXPHOS activity, in order to meet extra energy requirements beyond the basal levels.

To elucidate whether this deficiency was related with altered mitochondrial complexes, we evaluated the expression levels of complex IV (COX IV). COX IV is a mitochondrial enzyme, which has previously been shown to be highly affected by Aβ accumulation in other cell types [30]. Western blot analysis demonstrated that COX IV levels were significantly decreased in the astrocytes exposed to Aβ compared to control astrocytes, both at 7d and 7d + 6d (Fig. 6H). Interestingly, the expression levels of VDAC-1, an important mitochondrial Ca^2+^ channel, remained stable (Fig. 6I). Immunocytochemistry of Aβ-exposed astrocytes and control astrocytes showed that COX IV and VDAC-1 did not fully overlap, suggesting that there is a mitochondrial population that is COX IV negative (Fig. 6J). In addition, the mitochondrial dysfunction in Aβ-exposed astrocytes did not affect the overall cell viability (Additional file 7B) or the percentage of apoptotic cells in the culture (Additional file 7C, D). These results suggest that, although Aβ accumulation causes mitochondrial deficits, during heavy workload (stimulated by FCCP), the ATP levels remained unchanged, indicating that the energy metabolism in the Aβ-exposed astrocytes is switched to a different pathway.

### Astrocytes increase glycolysis after Aβ exposure

**Fig. 7:**
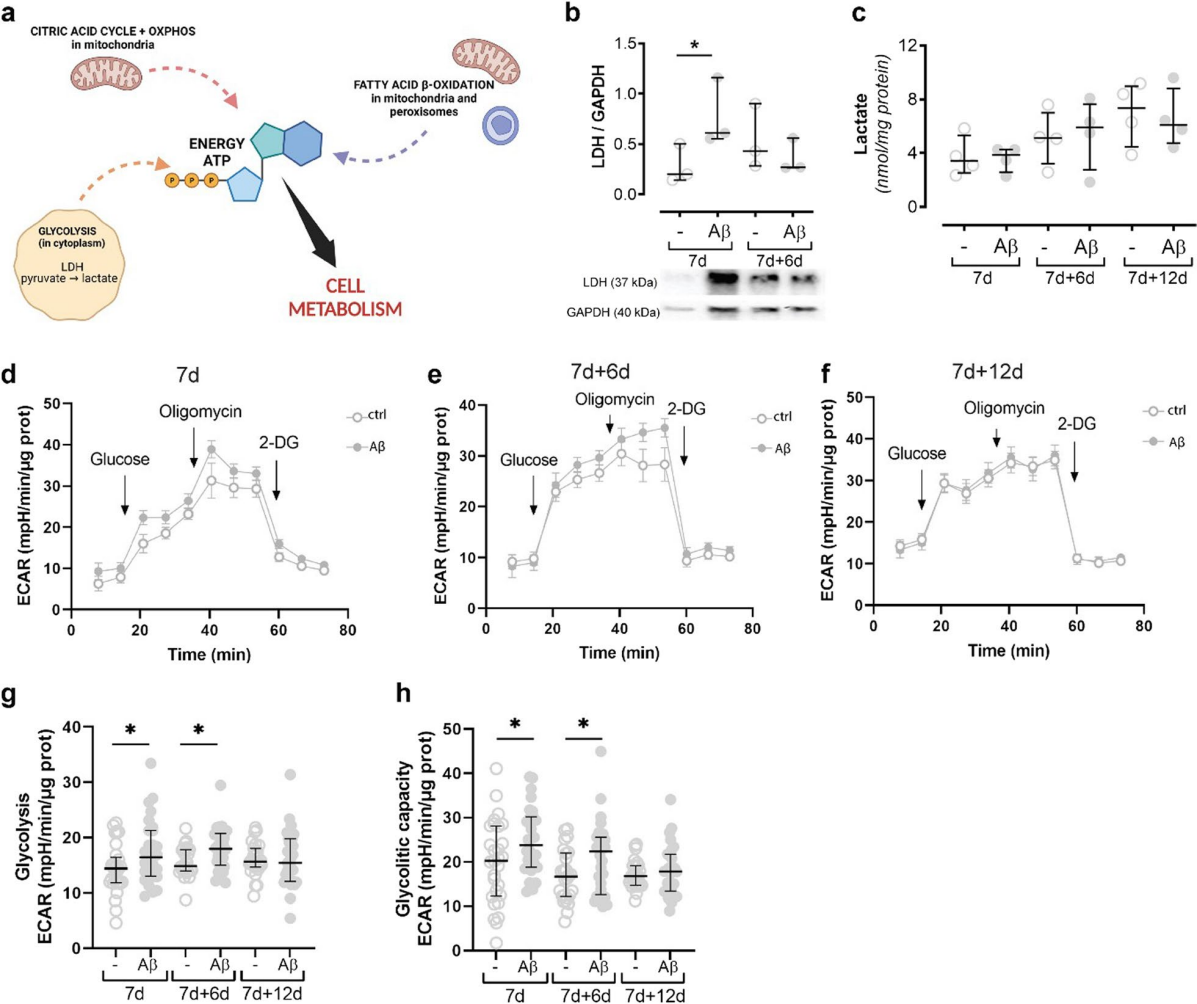
Glycolysis is increased in Aβ-accumulating astrocytes. There are three main pathways for energy production in cells: glycolysis, the citric acid cycle + OXPHOS, and fatty acid β oxidation (**A**). Although LDH levels were increased at 7 day after Aβ exposure (**B**), lactate levels remained stable (**C**), suggesting that the astrocytes try to switch to glycolysis but are not successful. Seahorse analysis revealed that both glycolysis and glycolytic capacity of Aβ-exposed astrocytes were increased at day 7 and 7 + 6 (**D**, **E**, **G**, **H**). This increase was, however, no longer observed at 7d + 12d (**F**–**H**)

There are three principal pathways for energy production in astrocytes: glycolysis, citric acid cycle coupled with OXPHOS, and fatty acid β oxidation (Fig. 7A). Because the astrocytes showed a decreased activity of OXPHOS following Aβ exposure, we next sought to investigate whether the glycolysis pathway was upregulated. Astrocytes are glycolytic cells and are known to convert pyruvate that results from glycolysis into lactate by the enzyme lactate dehydrogenase (LDH). Hence, we measured the levels of both LDH and lactate. The LDH levels increased significantly after 7d of Aβ exposure (Fig. 7B), suggesting that the astrocytes tried to use the glycolysis pathway for energy production. Surprisingly, lactate levels were unchanged (Fig. 7C), suggesting either dysfunctional glycolysis or higher rate conversion of lactate into pyruvate. To test that hypothesis, we used the Seahorse to evaluate proton production through ECAR, an indirect measurement of cellular glycolytic phenotype. We observed that both glycolysis and glycolytic capacity of Aβ-exposed astrocytes were increased at day 7 and 7 + 6 (Fig. 7D, E, G, H). This increase was no longer observed at 7d + 12d (Fig. 7F–H), when SRC was affected. Taken together, these data indicate that at 7d and 7d + 6d, OXPHOS activity in Aβ-exposed astrocytes was sustained by an increase in glycolysis.

### Energy production in Aβ-accumulating astrocytes switches to fatty acid β oxidation

**Fig. 8:**
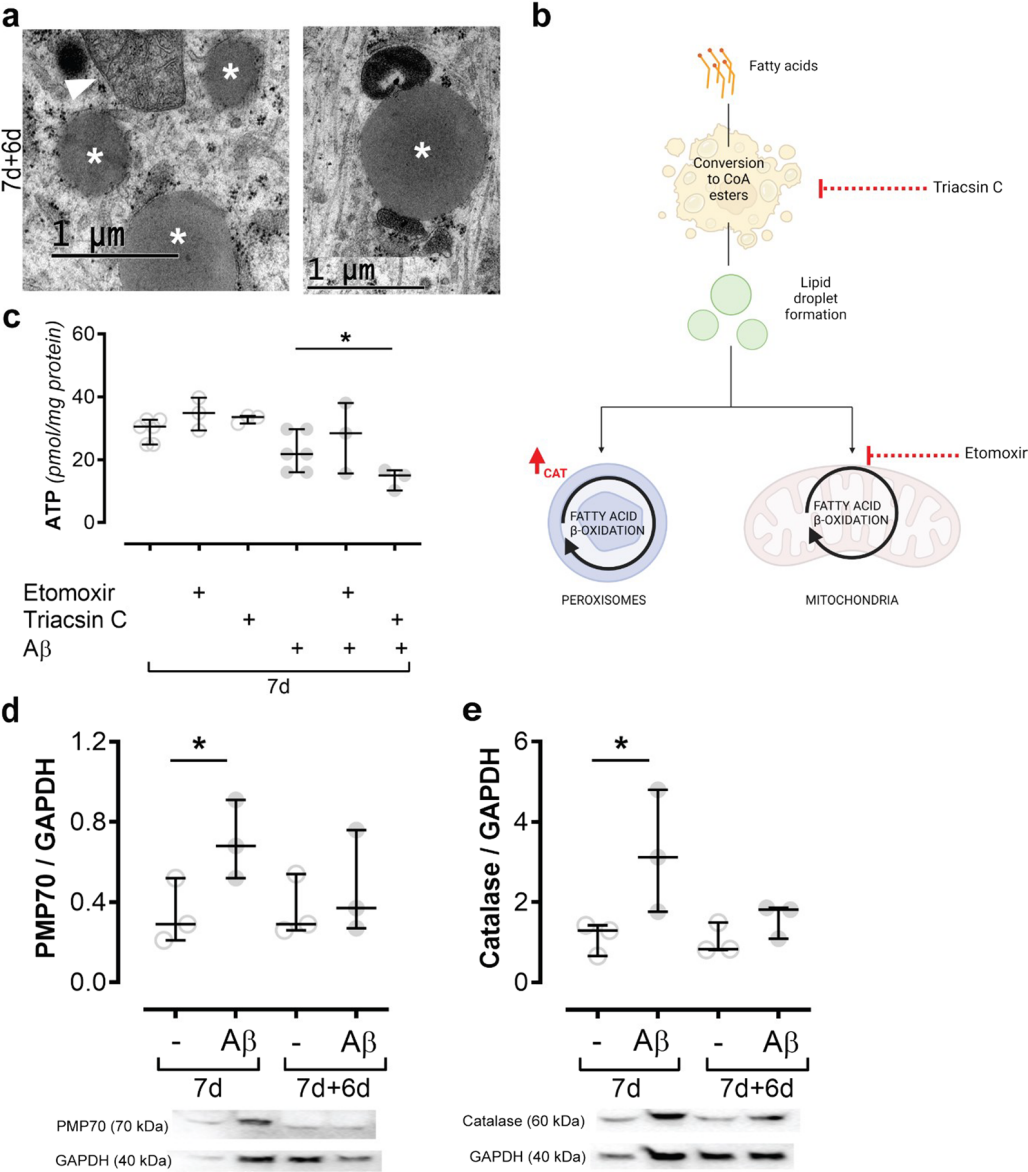
Astrocytes switch to fatty acid β oxidation for energy production after Aβ exposure. Lipid droplets (white asterisks) were observed in close proximity to mitochondria (white arrowhead) in Aβ-exposed astrocytes when TEM was performed (**A**). To further investigate fatty acid β oxidation the cells were treated with etomoxir, which inhibits the transport of fatty acids inside the mitochondria, or triacsin C, inhibiting the formation of neutral lipids from fatty acids (**B**). ATP levels were lowered when the astrocytes were exposed to both Aβ and triacsin C, suggesting that there is a switch to fatty acid β oxidation (**C**). Peroxisomes can also be a hub for fatty acid β oxidation and both the peroxisomal markers PMP70 and catalase show an increase 7 after the astrocytes were exposed to Aβ (**D**, **E**)

Interestingly, our TEM analysis of Aβ-accumulating astrocytes frequently showed lipid droplets in close proximity to mitochondria (Fig. 8A and Additional file 8), which may indicate that the droplets are used as a source for fatty acid β oxidation. The droplets themselves are not pathological structures and lipid droplets could also be found in control astrocytes (Additional file 8). To fully elucidate the energy metabolism in Aβ-exposed astrocytes, we next performed ATP-analysis experiments in the presence of two different inhibitors, specifically blocking certain stages of the fatty acid β oxidation pathway. Etomoxir inhibits the transport of fatty acids from the cytosol into the mitochondria, while triacsin C inhibits the de novo synthesis of neutral lipids from fatty acids and the formation of lipid droplets (Fig. 8B). When adding etomoxir to the Aβ-exposed astrocytes, we did not detect any changes in ATP levels, suggesting that fatty acid β oxidation takes place not only in mitochondria. To confirm that fatty acid β oxidation is responsible for the ATP production, triacsin C was used to inhibit the formation of lipid droplets. Lipid droplets are characteristic lipid-rich organelles that are used by various cell types for lipid storage and fatty acid β oxidation. When the astrocytes were exposed to triacsin C, the ATP levels were clearly reduced (Fig. 8C) in Aβ-exposed astrocytes, but not in control astrocytes, pointing to a switch towards fatty acid β oxidation for energy production. Besides mitochondria, fatty acid β oxidation takes place in peroxisomes. Therefore, we examined the peroxisome markers PMP70, which is situated in the peroxisomal membrane, and catalase, which is directly involved in peroxisomal β-oxidation. After 7d of Aβ-exposure, both markers were clearly increased (Fig. 8D, E), suggesting an increase in peroxisomal biogenesis. Consequently, our results support that astrocytes change their energy production pathway towards fatty acid β oxidation in response to Aβ accumulation. The initial increment of glycolysis and fatty acid β oxidation after Aβ exposure may consist in a cellular mechanism to sustain energy metabolism, explaining why deficits in mitochondrial respiration only occur 12 days after Aβ removal.

## Discussion

Growing evidence indicates that astrocytes play a central role in Aβ-mediated pathology and may constitute a strategic treatment target. Our previous data demonstrates that astrocytes ingest and store large deposits of aggregated Aβ. A high proportion of the astrocytic Aβ is N-terminally truncated [20]. Hence, the Aβ is processed and modified by the astrocytes, although not completely digested. This incomplete degradation results in the release of EVs containing neurotoxic Aβ species and increased levels of apoE [18, 20, 31]. More specifically, our data demonstrates that the Aβ-EVs induce synaptic loss, axonal swelling and vacuolization of the neuronal cell bodies, which consequently lead to apoptosis [18]. In this study, we aimed to investigate how the Aβ accumulation affect the astrocytes themselves, focusing on their energy metabolism. The human brain is a high-energy consuming organ. The energy demand is particularly pronounced in the neurons [32], although astrocytes have the foremost responsibility for the energy metabolism.

Mitochondria, the power stations of the cell, are very dynamic organelles. Their number is controlled through fission and fusion, because they cannot be generated de novo [33]. During fusion, the mitochondria mix their compartments to ensure that the DNA is inherited throughout the mitochondrial population. Fission increases the mitochondria number, but is also important for the distribution of mitochondria in the cell [33]. Our results suggest that Aβ-accumulation in astrocytes causes a disruption of mitochondrial dynamics. More specifically, analysis of the mitochondria motility revealed that in Aβ-exposed astrocytes the mitochondria move significantly longer distances/time than in control astrocytes. In addition, Aβ accumulation causes an imbalance in fission and fusion that changes over time. Six days after Aβ withdrawal, there was a clear decrease in the number of mitochondria, due to increased fusion or ineffective fission. This finding was confirmed by TEM, which showed very long and abnormally branched mitochondria in the Aβ-accumulating astrocytes. Twelve days after Aβ withdrawal, the number of mitochondria was instead increased, indicating excessive mitochondrial fission. This result was verified by TEM, demonstrating that the Aβ-accumulating astrocytes had multiple very short and swollen mitochondria. When recruited to the mitochondrial membrane, DRP-1 is responsible for mitochondrial fission. This is a well-controlled process, that DRP-1 can be either trigger or inhibit, depending on which sites of the protein that is phosphorylated (at serine 616 (S616) or serine 637 (S637), respectively) [28, 29]. It has previously been shown that there is an overall decrease of DRP-1 protein in the AD brain, but an increase in pDRP-1 (S616) [34]. Here, we found that astrocytes had elevated levels of pDRP-1 (S616) directly after the 7 day Aβ exposure and 6 days later there was an increase in pDRP-1 (S637). It is possible that DRP-1, in addition to its role in fission regulation, also have other functions in the cell that are not mitochondria related. For example, it has been shown that DRP-1 can affect synapse formation and embryonic development [35], postsynaptic endocytosis and dendritic growth in neurons [36], peroxisomal fission [37, 38] and LDs dissociation from the ER [39]. Notably, our immunostainings revealed that the pattern of pDRP-1 (S616) changed dramatically following Aβ exposure. The Aβ-accumulating astrocytes displayed large deposits of pDRP-1 (S637) that co-localized with BODIPY-positive lipid droplets. Interestingly, we could demonstrate secretion of the modified DRP-1 forms to the media and inter-cellular transfer of the pDRP-1 (S616) deposits from one astrocyte to another via TNTs and thicker protrusions. In line with our findings, a recent study suggests an integral role of DRP-1 in fatty acid β oxidation [40]. The authors demonstrated that DRP-1 knock-out cell line translocate LDs to their mitochondria, but have a decreased fatty acid degradation. To fully understand the relevance of DRP-1 for cell metabolism, additional studies will be needed, but it is clear that DRP-1 has multiple functions in human astrocytes that are affected by the presence of Aβ pathology.

Energy metabolism is vital for cell functions, and there are several pathways to produce energy: glycolysis, the citric acid cycle followed by OXPHOS, and fatty acid β metabolism. The citric acid cycle with OXPHOS is a very effective pathway for energy production and takes place in the matrix respective the inner membrane of mitochondria [41]. Our Seahorse flux OCR analysis showed that maximal respiration and SCR were severely affected in Aβ-exposed astrocytes, 12 days after Aβ withdrawal, suggesting that astrocytes cannot increase OXPHOS activity under stress in the long run. COX IV is an enzyme involved in the electron transport chain and in OXPHOS and is therefore a key player in energy production. Our result revealed a decrease in COX IV levels in astrocytes exposed to Aβ that was not a result of decreased mitochondrial number, measured by the mitochondrial membrane marker VDAC-1. These results suggest that parts of the OXPHOS pathways are dysregulated by Aβ exposure, which consequently could result in less ATP production. Interestingly ATP levels remained unchanged in the Aβ-exposed astrocytes, indicating that the astrocytes switched to a different pathway for energy production. This was also supported by the fact that astrocytic viability was unaffected by the Aβ-exposure, based on the apoptotic markers caspase-3 and BAX. It has been shown that Aβ induces energy production in microglia to shift from OXPHOS to glycolysis [42], therefore we investigated if this was also the case in the astrocytic cultures. Astrocytes are glycolytic cells that have lactate as an end-product of glycolysis instead of pyruvate [7]. We analyzed the levels of LDH, the enzyme that converts pyruvate into lactate, and lactate in order to investigate if the energy production had switched to glycolysis. We found that the LDH levels rose directly after the 7 days of Aβ-exposure, suggesting that the astrocytes tried to increase the glycolysis. However, lactate levels remained unchanged, pointing at either dysfunctional glycolysis or at a higher conversion rate of lactate into pyruvate. Both glycolysis and glycolytic capacity were increased in Aβ accumulating astrocytes directly after Aβ exposure and 6 days later, but not at the latest time point. These results suggest that glycolysis support OXPHOS at the earlier time points, but not continuously.

During starvation and stress astrocytes have been reported to increase the formation of LDs [9, 43]. In Aβ-accumulating astrocytes, we observed LDs in close proximity to mitochondria, suggesting an interaction between the two. Lipid droplets can provide mitochondria with fatty acids for fatty acid β oxidation. To elucidate if energy production in the astrocytes switched to fatty acid β oxidation, we used two inhibitors: etomoxir and triacsin C. Etomoxir inhibits the shuttling of fatty acids inside the mitochondria, while triacsin C inhibits the production of neutral lipids from fatty acids needed for the formation of LDs. The combination of triacsin C and Aβ exposure caused a drop in ATP levels, suggesting that LDs play a role in the astrocytes’ energy production under pathogenic conditions. When the astrocytes were treated with etomoxir, the ATP levels remained stable, indicating that fatty acid β oxidation does not after all take place in the mitochondria. Another organelle, the peroxisome, is also able to oxidize fatty acids to produce energy [44] and has been shown to interact with LDs [45]. This switch to peroxisomal fatty acid β oxidation was confirmed by increased levels of the peroxisomal markers PMP70 and catalase. A previous study has shown that peroxisomal proliferation protects neurons from Aβ toxicity [46]. Our results suggest that the increase of peroxisomal activity has a similar protective role in astrocytes.

## Conclusions

Alzheimer’s disease is the leading cause of dementia. Yet, no treatments that limit neurodegeneration or slow down the progression of the disease are available. In order to identify novel treatment targets, detailed knowledge about the mechanisms of AD progression is highly desirable. According to the amyloid cascade hypothesis, Aβ accumulation is the causative agent of AD, consequently driving the formation of neurofibrillary tangles, inflammation and finally neuronal cell loss. However, the exact cellular and molecular mechanisms by which Aβ induce these processes remain unclear. Here, we show that that Aβ accumulation severely affects the mitochondria in human astrocytes. However, by shifting their energy production to peroxisomal fatty acid β-oxidation, the astrocytes can maintain functionality and keep constant ATP levels. In the brain, such a maneuver by the astrocytes would have repercussions for their capability to maintain homeostasis and support neuronal energy metabolism, which may accelerate the disease course.

## Supplementary Information

**Additional file 1. **Astrocytic markers. hiPSC-derived astrocytes were stained with DAPI (blue) and the astrocytic markers: GLAST-1 (A), Vimentin (B) S100β (C), AQP4 (D), GFAP (E), and Nestin (F). Scale bar: 20 μm.

**Additional file 2. **Aβ exposure affects mitochondrial motility in astrocytes. The parameter displacement, distance, velocity and speed were analyzed with Mitometer (A). Motility analysis showed a significant increase in the mitochondrial speed (B) and velocity (C) in Aβ-exposed astrocytes.

**Additional file 3. **Mitochondrial fusion protein levels are not affected by Aβ exposure. Western blot analysis demonstrated that OPA1 levels remain unchanged by Aβ exposure at 7d and 7d+6d.

**Additional file 4. **pDRP-1 (S637) encapsulated BODIPY positive lipid droplets in Aβ exposed astrocytes. Lipid droplets (b, green) in Aβ exposed astrocytes are surrounded by pDRP-1 (S637) (c, red). Scale bar = 2 μm.

**Additional file 5. **Mitochondria are distributed between astrocytes via tunneling nanotubes. Arrows indicate transfer of MitoTracker™ Green labeled mitochondria from the top cell to the bottom cell via tunneling nanotubes. The top panel show fluorescent mitochondria in white and the bottom panel show the merged images (GFP and bright field) of the same time points.

**Additional file 6. **Cell to cell mitochondrial transfer. The movie shows a time-lapse recording of MitoTracker™ Green labeled astrocytes. Images were captured at 20x every 2 minutes.

**Additional file 7. **Aβ exposure does not affect basal respiration of mitochondria, cell viability or apoptosis in astrocytes. Seahorse OCR analysis showed no differences in mitochondrial basal respiration between control and Aβ-exposed astrocytes (A). Alamar blue assay showed no decrease in viability in the astrocyte cultures exposed to Aβ (B). Similarly, WB analysis showed no increase in apoptosis markers caspase-3 and BAX in the Aβ-exposedcultures (C and D).

**Additional file 8. **Lipid droplets are found in both Aβ exposed and control astrocytes. Lipid droplets (white asterisks) are observed in Aβ exposed astrocytes (a,b) and in controls (c,c´).

## Data Availability

The datasets used and/or analyzed during the current study are available from the corresponding author on reasonable request.

## Abbreviations

Aβ: Amyloid-beta
AD: Alzheimer’s disease
Ant A: Antimycin A
bFGF: Basic fibroblast growth factor
BSA: Bovine serum albumin
CNTF: Ciliary neurotrophic factor
COX IV: Complex IV
DRP-1: Dynamin related protein 1
ECAR: Extracellular acidification rate
ECL: Enhanced chemiluminescence
ER: Endoplasmic reticulum
EVs: Extracellular vesicles
FCCP: Carbonyl cyanide-4-(trifluoromethoxy)phenylhydrazone
hiPSC: Human induced pluripotent cell
HRP: Horseradish peroxidase-conjugated
ICC: Immunocytochemistry
IGF-1: Insulin-like growth factor 1
LD: Lipid droplet
LDH: Lactate dehydrogenase
MHCII: Major histocompatibility complex class II
NES: Neuroepithelial-like stem
NGS: Normal goat serum
OCR: Oxygen consumption rate
OXPHOS: Oxidative phosphorylation
PFA: Paraformaldehyde
Rot: Rotenone
SDS: Sodium dodecyl sulfate
SRC: Spare respiratory capacity
TBS-T: Tris-buffered saline-Tween
TEM: Transmission electron microscopy
TNTs: Tunneling nanotubes
VDAC-1: Voltage-dependent anion-selective channel 1
WB: Western blot

## References

[1] Serrano-Pozo A, Frosch MP, Masliah E, Hyman BT (2011). Neuropathological alterations in Alzheimer disease. Cold Spring Harb Perspect Med.

[2] Abbott NJ, Rönnbäck L, Hansson E (2006). Astrocyte–endothelial interactions at the blood–brain barrier. Nat Rev Neurosci.

[3] Allen NJ (2014). Astrocyte regulation of synaptic behavior. Annu Rev Cell Dev Biol.

[4] Hablitz LM, Plá V, Giannetto M, Vinitsky HS, Stæger FF, Metcalfe T (2020). Circadian control of brain glymphatic and lymphatic fluid flow. Nat Commun.

[5] MacVicar BA, Newman EA (2015). Astrocyte regulation of blood flow in the Brain. Cold Spring Harb Perspect Biol.

[6] Vasile F, Dossi E, Rouach N (2017). Human astrocytes: structure and functions in the healthy brain. Brain Struct Funct.

[7] Barros LF, San Martín A, Ruminot I, Sandoval PY, Baeza-Lehnert F, Arce-Molina R (2020). Fluid brain glycolysis: limits, speed, location, moonlighting, and the fates of glycogen and lactate. Neurochem Res.

[8] Koopman WJH, Distelmaier F, Smeitink JAM, Willems PHGM (2013). OXPHOS mutations and neurodegeneration. EMBO J.

[9] Rambold AS, Cohen S, Lippincott-Schwartz J (2015). Fatty acid trafficking in starved cells: regulation by lipid droplet lipolysis, autophagy, and mitochondrial fusion dynamics. Dev Cell.

[10] Mulica P, Grünewald A, Pereira SL (2021). Astrocyte-neuron metabolic crosstalk in neurodegeneration: a mitochondrial perspective. Front Endocrinol (Lausanne).

[11] Mecocci P, Baroni M, Senin U, Boccardi V (2018). Brain aging and late-onset Alzheimer’s disease: a matter of increased amyloid or reduced energy?. J Alzheimers Dis.

[12] Ralhan I, Chang CL, Lippincott-Schwartz J, Ioannou MS (2021). Lipid droplets in the nervous system. J Cell Biol.

[13] Nagele RG, D’Andrea MR, Lee H, Venkataraman V, Wang HY (2003). Astrocytes accumulate A beta 42 and give rise to astrocytic amyloid plaques in Alzheimer disease brains. Brain Res.

[14] Gomez-Arboledas A, Davila JC, Sanchez-Mejias E, Navarro V, Nuñez-Diaz C, Sanchez-Varo R (2018). Phagocytic clearance of presynaptic dystrophies by reactive astrocytes in Alzheimer’s disease. Glia.

[15] Thal DR, Schultz C, Dehghani F, Yamaguchi H, Braak H, Braak E (2000). Amyloid beta-protein (Abeta)-containing astrocytes are located preferentially near N-terminal-truncated Abeta deposits in the human entorhinal cortex. Acta Neuropathol.

[16] Funato H, Yoshimura M, Yamazaki T, Saido TC, Ito Y, Yokofujita J (1998). Astrocytes containing amyloid beta-protein (Abeta)-positive granules are associated with Abeta40-positive diffuse plaques in the aged human brain. Am J Pathol.

[17] Maat-Schieman MLC, Yamaguchi H, Hegeman-Kleinn IM, Welling-Graafland C, Natté R, Roos RAC (2004). Glial reactions and the clearance of amyloid β protein in the brains of patients with hereditary cerebral hemorrhage with amyloidosis-Dutch type. Acta Neuropathol.

[18] Beretta C, Nikitidou E, Streubel-Gallasch L, Ingelsson M, Sehlin D, Erlandsson A (2020). Extracellular vesicles from amyloid-β exposed cell cultures induce severe dysfunction in cortical neurons. Sci Rep.

[19] Rostami J, Mothes T, Kolahdouzan M, Eriksson O, Moslem M, Bergström J (2021). Crosstalk between astrocytes and microglia results in increased degradation of α-synuclein and amyloid-β aggregates. J Neuroinflammation.

[20] Söllvander S, Nikitidou E, Brolin R, Söderberg L, Sehlin D, Lannfelt L (2016). Accumulation of amyloid-β by astrocytes result in enlarged endosomes and microvesicle-induced apoptosis of neurons. Mol Neurodegener.

[21] Rostami J, Holmqvist S, Lindström V, Sigvardson J, Westermark GT, Ingelsson M (2017). Human astrocytes transfer aggregated alpha-synuclein via tunneling nanotubes. J Neurosci.

[22] Rostami J, Fotaki G, Sirois J, Mzezewa R, Bergström J, Essand M (2020). Astrocytes have the capacity to act as antigen-presenting cells in the Parkinson’s disease brain. J Neuroinflammation.

[23] Falk A, Koch P, Kesavan J, Takashima Y, Ladewig J, Alexander M (2012). Capture of neuroepithelial-like stem cells from pluripotent stem cells provides a versatile system for in vitro production of human neurons. PLoS ONE.

[24] Lundin A, Delsing L, Clausen M, Ricchiuto P, Sanchez J, Sabirsh A (2018). Human iPS-derived astroglia from a stable neural precursor state show improved functionality compared with conventional astrocytic models. Stem Cell Reports.

[25] Lefebvre AEYT, Ma D, Kessenbrock K, Lawson DA, Digman MA (2021). Automated segmentation and tracking of mitochondria in live-cell time-lapse images. Nat Methods.

[26] Valente AJ, Maddalena LA, Robb EL, Moradi F, Stuart JA (2017). A simple ImageJ macro tool for analyzing mitochondrial network morphology in mammalian cell culture. Acta Histochem.

[27] Streubel-Gallasch L, Giusti V, Sandre M, Tessari I, Plotegher N, Giusto E (2021). Parkinson’s disease-associated LRRK2 interferes with astrocyte-mediated alpha-synuclein clearance. Mol Neurobiol.

[28] Ko AR, Hyun HW, Min SJ, Kim JE (2016). The differential DRP1 phosphorylation and mitochondrial dynamics in the regional specific astroglial death induced by status epilepticus. Front Cell Neurosci.

[29] Ko HJ, Tsai CY, Chiou SJ, Lai YL, Wang CH, Cheng JT (2021). The Phosphorylation Status of Drp1-Ser637 by PKA in Mitochondrial Fission Modulates Mitophagy via PINK1/Parkin to Exert Multipolar Spindles Assembly during Mitosis. Biomolecules.

[30] Casley CS, Canevari L, Land JM, Clark JB, Sharpe MA (2002). β-Amyloid inhibits integrated mitochondrial respiration and key enzyme activities. J Neurochem.

[31] Nikitidou E, Khoonsari PE, Shevchenko G, Ingelsson M, Kultima K, Erlandsson A (2017). Increased release of apolipoprotein e in extracellular vesicles following amyloid-β protofibril exposure of neuroglial co-cultures. J Alzheimers Dis.

[32] Deitmer JW, Theparambil SM, Ruminot I, Noor SI, Becker HM (2019). Energy dynamics in the brain: contributions of astrocytes to metabolism and pH homeostasis. Front Neurosci.

[33] Westermann B (2010). Mitochondrial fusion and fission in cell life and death. Nat Rev Mol Cell Biol.

[34] Wang X, Su B, Lee H, Li X, Perry G, Smith MA (2009). Impaired balance of mitochondrial fission and fusion in Alzheimer’s disease. J Neurosci.

[35] Ishihara N, Nomura M, Jofuku A, Kato H, Suzuki SO, Masuda K (2009). Mitochondrial fission factor Drp1 is essential for embryonic development and synapse formation in mice. Nat Cell Biol.

[36] Itoh K, Murata D, Kato T, Yamada T, Araki Y, Saito A (2019). Brain-specific Drp1 regulates postsynaptic endocytosis and dendrite formation independently of mitochondrial division. Life.

[37] Kamerkar SC, Kraus F, Sharpe AJ, Pucadyil TJ, Ryan MT (2018). Dynamin-related protein 1 has membrane constricting and severing abilities sufficient for mitochondrial and peroxisomal fission. Nat Commun.

[38] Koch A, Thiemann M, Grabenbauer M, Yoon Y, McNiven MA, Schrader M (2003). Dynamin-like Protein 1 Is Involved in Peroxisomal Fission*. J Biol Chem.

[39] Li X, Yang L, Mao Z, Pan X, Zhao Y, Gu X (2020). Novel role of dynamin-related-protein 1 in dynamics of ER-lipid droplets in adipose tissue. FASEB J.

[40] Xiong X, Hasani S, Young LEA, Rivas DR, Skaggs AT, Martinez R (2022). Activation of Drp1 promotes fatty acids-induced metabolic reprograming to potentiate Wnt signaling in colon cancer. Cell Death Differ.

[41] Nolfi-Donegan D, Braganza A, Shiva S (2020). Mitochondrial electron transport chain: Oxidative phosphorylation, oxidant production, and methods of measurement. Redox Biol.

[42] Baik SH, Kang S, Lee W, Choi H, Chung S, Kim JI (2019). A breakdown in metabolic reprogramming causes microglia dysfunction in Alzheimer’s Disease. Cell Metab.

[43] Smolič T, Tavčar P, Horvat A, Černe U, Halužan Vasle A, Tratnjek L (2021). Astrocytes in stress accumulate lipid droplets. Glia.

[44] Jo DS, Park NY, Cho DH (2020). Peroxisome quality control and dysregulated lipid metabolism in neurodegenerative diseases. Exp Mol Med.

[45] Valm AM, Cohen S, Legant WR, Melunis J, Hershberg U, Wait E (2017). Applying systems-level spectral imaging and analysis to reveal the organelle interactome. Nature.

[46] Santos MJ, Quintanilla RA, Toro A, Grandy R, Dinamarca MC, Godoy JA (2005). Peroxisomal proliferation protects from β-Amyloid neurodegeneration*. J Biol Chem.

