# Supplementary material for: Amyloid-β accumulation in human astrocytes induces mitochondrial disruption and changed energy metabolism
Source: J Neuroinflammation. 2023 Feb 20;20:43. doi: 10.1186/s12974-023-02722-z (PMC9940442; doi:10.1186/s12974-023-02722-z)
Supplement: Supplementary file 1 — Additional file 1. Astrocytic markers. hiPSC-derived astrocytes were stained with DAPI (blue) and the astrocytic markers: GLAST-1 (A), Vimentin (B) S100β (C), AQP4 (D), GFAP (E), and Nestin (F). Scale bar: 20 μm. [file 12974_2023_2722_MOESM1_ESM.pdf]

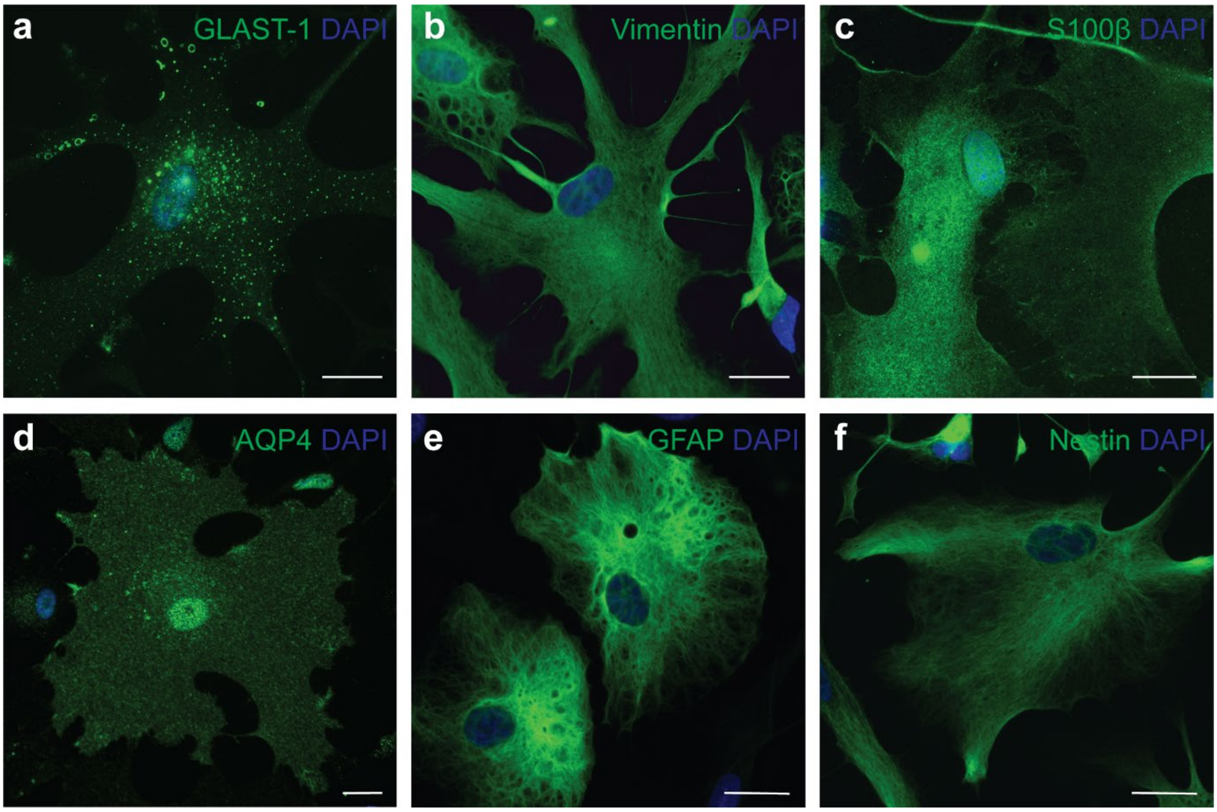

Additional file 1. Astrocytic markers. hiPSC-derived astrocytes were stained with DAPI (blue) and the astrocytic markers: GLAST-1 (A), Vimentin (B) S100β (C), AQP4 (D), GFAP (E), and Nestin (F). Scale bar: 20 µm.
