# Supplementary material for: Amyloid-β accumulation in human astrocytes induces mitochondrial disruption and changed energy metabolism
Source: J Neuroinflammation. 2023 Feb 20;20:43. doi: 10.1186/s12974-023-02722-z (PMC9940442; doi:10.1186/s12974-023-02722-z)
Supplement: Supplementary file 2 — Additional file 2. Aβ exposure affects mitochondrial motility in astrocytes. The parameter displacement, distance, velocity and speed were analyzed with Mitometer (A). Motility analysis showed a significant increase in the mitochondrial speed (B) and velocity (C) in Aβ-exposed astrocytes. [file 12974_2023_2722_MOESM2_ESM.pdf]

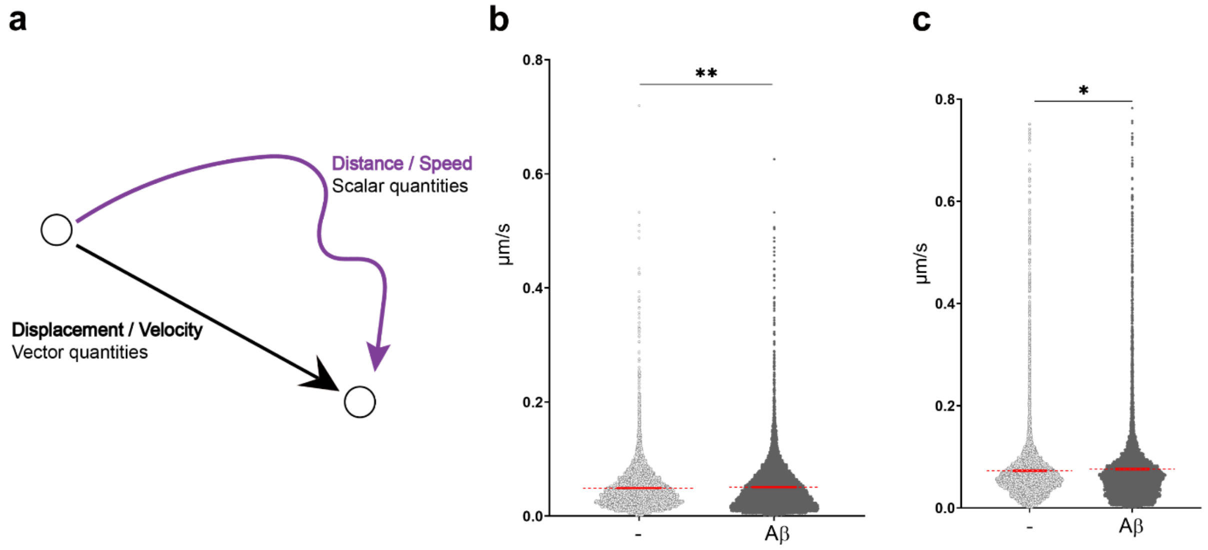

Additional file 2. Aβ exposure affects mitochondrial motility in astrocytes. The parameter displacement, distance, velocity and speed were analyzed with Mitometer (A). Motility analysis showed a significant increase in the mitochondrial speed (B) and velocity (C) in Aβ-exposed astrocytes.
