# Supplementary material for: Amyloid-β accumulation in human astrocytes induces mitochondrial disruption and changed energy metabolism
Source: J Neuroinflammation. 2023 Feb 20;20:43. doi: 10.1186/s12974-023-02722-z (PMC9940442; doi:10.1186/s12974-023-02722-z)
Supplement: Supplementary file 3 — Additional file 3. Mitochondrial fusion protein levels are not affected by Aβ exposure. Western blot analysis demonstrated that OPA1 levels remain unchanged by Aβ exposure at 7d and 7d+6d. [file 12974_2023_2722_MOESM3_ESM.pdf]

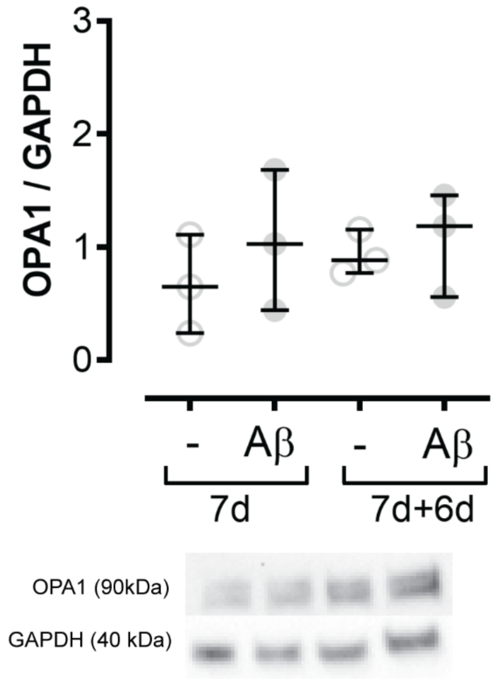

Additional file 3. Mitochondrial fusion protein levels are not affected by Aβ exposure. Western blot analysis demonstrated that OPA1 levels remain unchanged by Aβ exposure at 7d and 7d+6d.
