# Supplementary material for: Amyloid-β accumulation in human astrocytes induces mitochondrial disruption and changed energy metabolism
Source: J Neuroinflammation. 2023 Feb 20;20:43. doi: 10.1186/s12974-023-02722-z (PMC9940442; doi:10.1186/s12974-023-02722-z)
Supplement: Supplementary file 4 — Additional file 4. pDRP-1 (S637) encapsulated BODIPY positive lipid droplets in Aβ exposed astrocytes. Lipid droplets (b, green) in Aβ exposed astrocytes are surrounded by pDRP-1 (S637) (c, red). Scale bar = 2 μm. [file 12974_2023_2722_MOESM4_ESM.pdf]

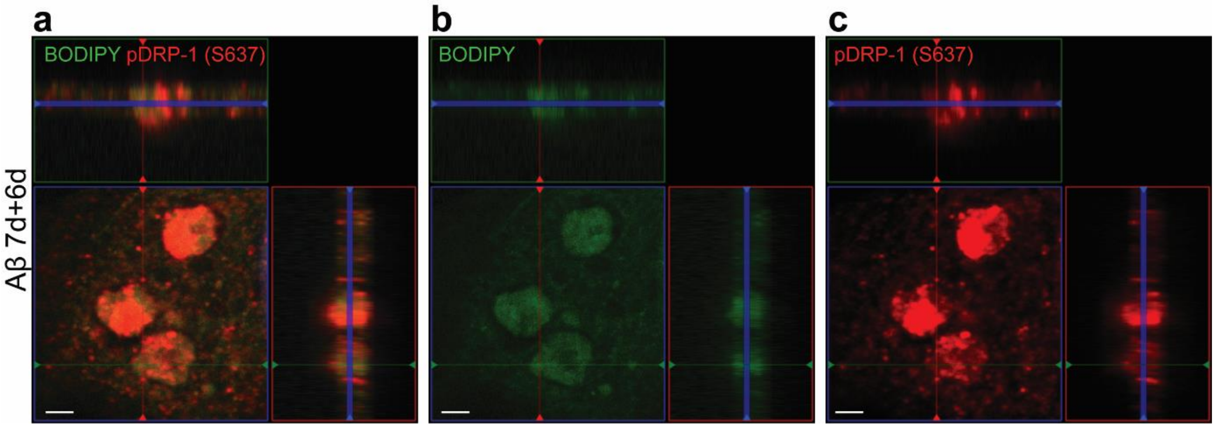

Additional file 4. pDRP-1 (S637) encapsulated BODIPY positive lipid droplets in Aβ exposed astrocytes. Lipid droplets (b, green) in Aβ exposed astrocytes are surrounded by pDRP-1 (S637) (c, red). Scale bar = 2 µm.
