# Supplementary material for: Amyloid-β accumulation in human astrocytes induces mitochondrial disruption and changed energy metabolism
Source: J Neuroinflammation. 2023 Feb 20;20:43. doi: 10.1186/s12974-023-02722-z (PMC9940442; doi:10.1186/s12974-023-02722-z)
Supplement: Supplementary file 5 — Additional file 5. Mitochondria are distributed between astrocytes via tunneling nanotubes. Arrows indicate transfer of MitoTracker™ Green labeled mitochondria from the top cell to the bottom cell via tunneling nanotubes. The top panel show fluorescent mitochondria in white and the bottom panel show the merged images (GFP and bright field) of the same time points. [file 12974_2023_2722_MOESM5_ESM.pdf]

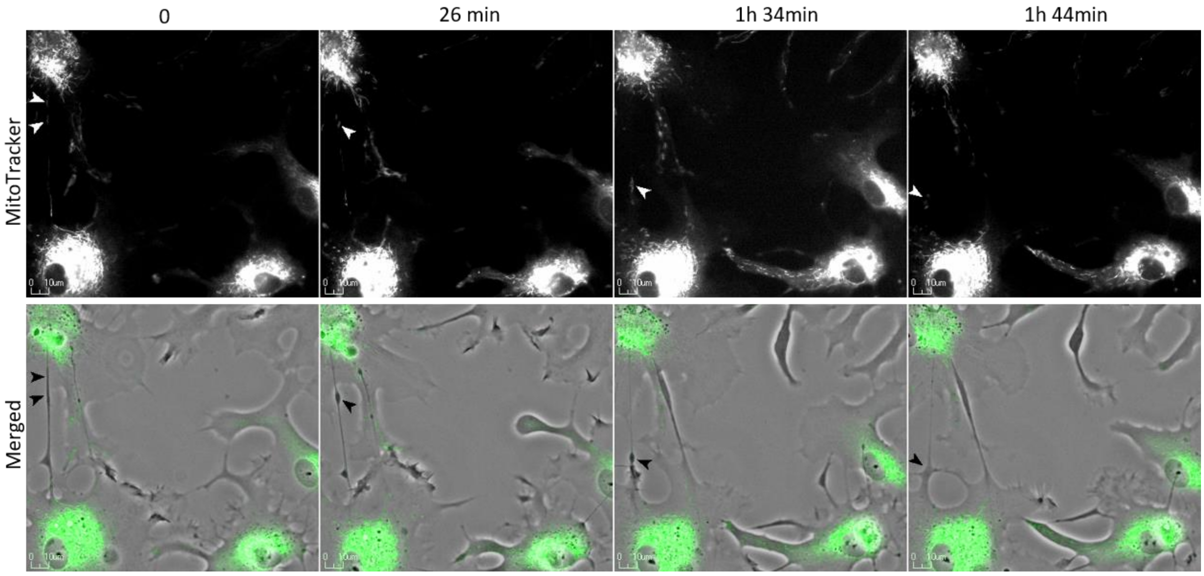

Additional file 5. Mitochondria are distributed between astrocytes via tunneling nanotubes. Arrows indicate transfer of MitoTracker™ Green labeled mitochondria from the top cell to the bottom cell via tunneling nanotubes. The top panel show fluorescent mitochondria in white and the bottom panel show the merged images (GFP and bright field) of the same time points.
