# Supplementary material for: Amyloid-β accumulation in human astrocytes induces mitochondrial disruption and changed energy metabolism
Source: J Neuroinflammation. 2023 Feb 20;20:43. doi: 10.1186/s12974-023-02722-z (PMC9940442; doi:10.1186/s12974-023-02722-z)
Supplement: Supplementary file 7 — Additional file 7. Aβ exposure does not affect basal respiration of mitochondria, cell viability or apoptosis in astrocytes. Seahorse OCR analysis showed no differences in mitochondrial basal respiration between control and Aβ-exposed astrocytes (A). Alamar blue assay showed no decrease in viability in the astrocyte cultures exposed to Aβ (B). Similarly, WB analysis showed no increase in apoptosis markers caspase-3 and BAX in the Aβ-exposedcultures (C and D). [file 12974_2023_2722_MOESM7_ESM.pdf]

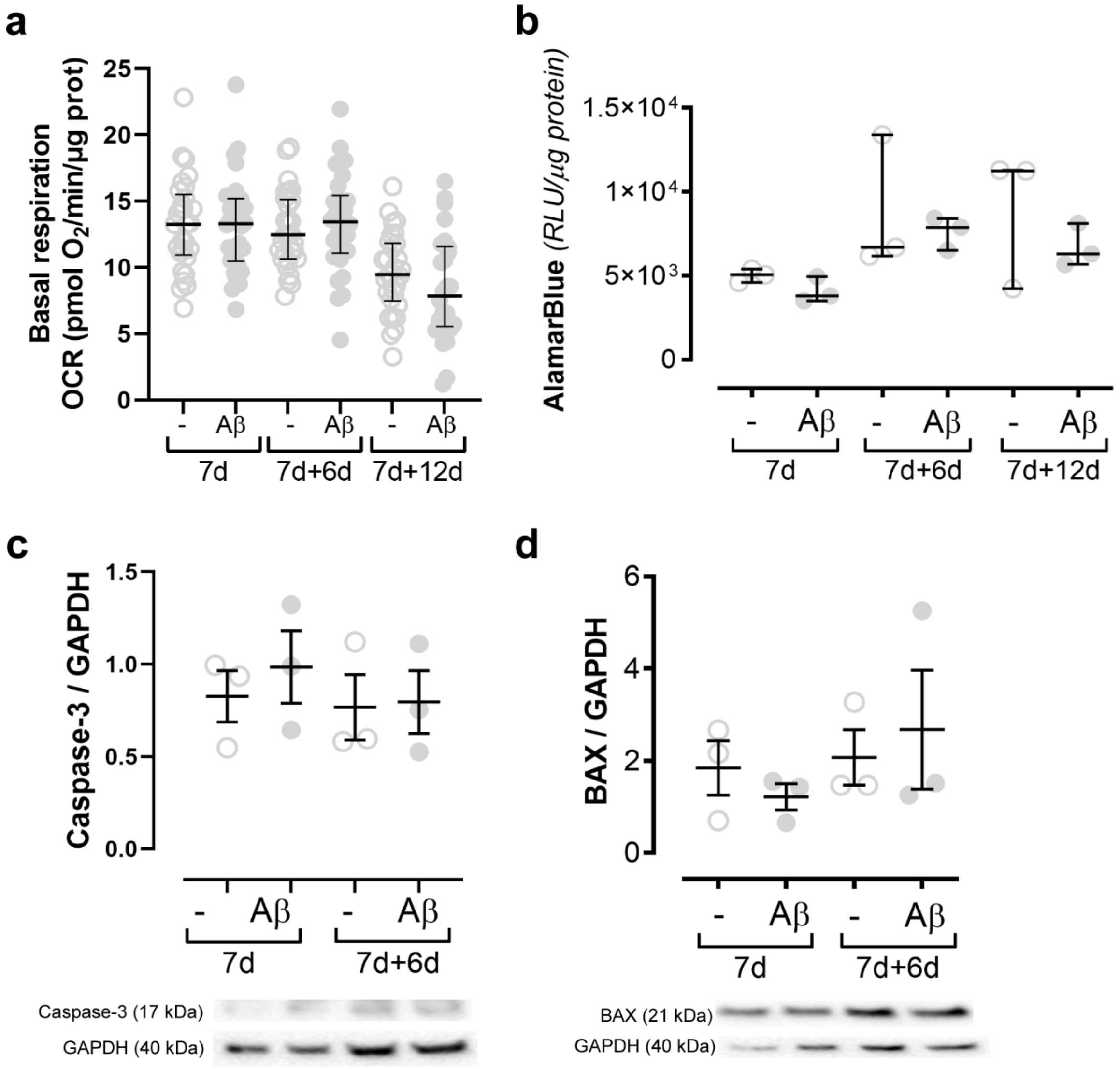

Additional file 7. Aβ exposure does not affect basal respiration of mitochondria, cell viability or apoptosis in astrocytes. Seahorse OCR analysis showed no differences in mitochondrial basal respiration between control and Aβ-exposed astrocytes (A). Alamar blue assay showed no decrease in viability in the astrocyte cultures exposed to Aβ (B). Similarly, WB analysis showed no increase in apoptosis markers caspase-3 and BAX in the Aβ-exposed cultures (C and D).
