# Supplementary material for: Amyloid-β accumulation in human astrocytes induces mitochondrial disruption and changed energy metabolism
Source: J Neuroinflammation. 2023 Feb 20;20:43. doi: 10.1186/s12974-023-02722-z (PMC9940442; doi:10.1186/s12974-023-02722-z)
Supplement: Supplementary file 8 — Additional file 8. Lipid droplets are found in both Aβ exposed and control astrocytes. Lipid droplets (white asterisks) are observed in Aβ exposed astrocytes (a,b) and in controls (c,c´). [file 12974_2023_2722_MOESM8_ESM.pdf]

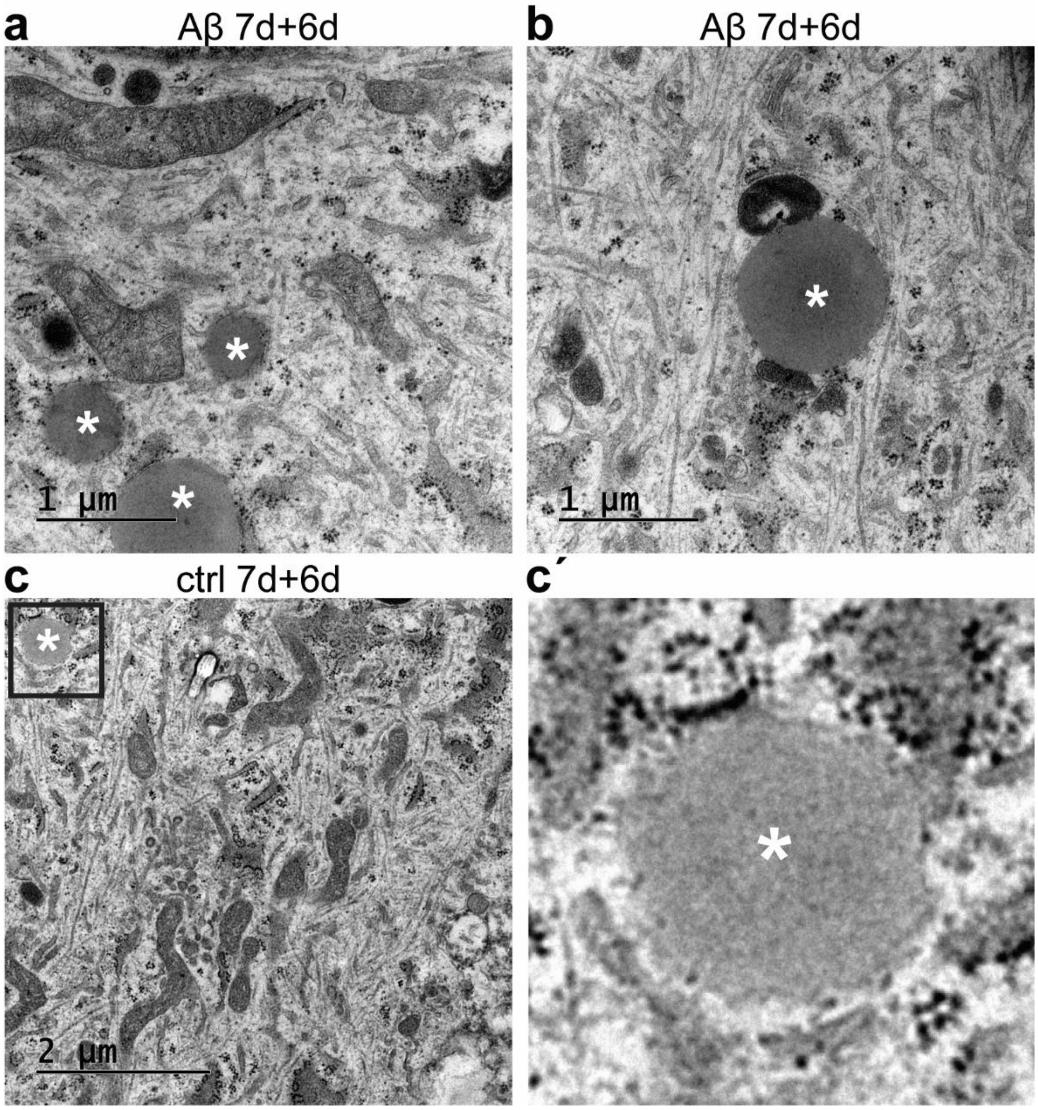

Additional file 8. Lipid droplets are found in both Aβ exposed and control astrocytes. Lipid droplets (white asterisks) are observed in Aβ exposed astrocytes (a,b) and in controls (c,c´).
